# Assessing the effectiveness of healthy weight interventions in the early years of childhood: a systematic review and meta-analysis of evidence from high-income countries

**DOI:** 10.1038/s41366-026-02073-8

**Published:** 2026-04-09

**Authors:** Disha Dhar, Lucy Karwatowska, Maria Sifaki, Semina Michalopoulou, Claire Stansfield, Jessica Packer, Simon J. Russell

**Affiliations:** 1 University College London, Great Ormond Street Institute of Child Health, London, UK; 2 University College London, Institute of Epidemiology and Health Care, London, UK; 3 University College London, Institute of Education, London, UK

**Keywords:** Epidemiology, Disease prevention, Health policy

## Abstract

**Background:**

Early childhood represents an important opportunity for establishing lifelong health behaviours, including the maintenance of a healthy weight. Given that weight patterns established in early life often persist into adolescence and adulthood, there is an urgent need to understand whether universal interventions in early childhood are effective in preventing excess weight gain. We aimed to systematically synthesise the effects of universal healthy weight interventions for pre-school children on anthropometric outcomes.

**Methods:**

We searched nine academic databases and included studies published from 2011 with pre-post implementation assessments of anthropometric outcomes amongst preschool children aged 5 years and under. Eligible studies included interventions that incorporated diet, physical activity, and/or behavioural change components. All studies were included in a narrative synthesis and, where possible, three-level random-effects meta-analyses were conducted to pool standardised mean differences.

**Results:**

A total of 40 studies (*n* = 26,977 participants) met the inclusion criteria. The most commonly reported outcomes were BMI *z*-score (zBMI; *k* [number of studies] = 27), weight status categories (*k* = 19), BMI (*k* = 14), BMI percentiles (*k* = 11), body weight (*k* = 8), waist circumference (*k* = 8), skinfolds (*k* = 6), percentage body fat (*k* = 3), fat-free mass index (*k* = 3), and fat mass index (*k* = 2). The findings from the meta-analyses suggested that children receiving interventions had significantly lower zBMI (SMD [standardised mean difference] = −0.085, 95% CI −0.140, −0.029), lower waist circumference (SMD = −0.186, 95% CI −0.361, −0.011), percentage body fat (SMD = −0.159, 95% CI −0.290, −0.028) and higher fat-free mass index (SMD = 0.170, 95% CI 0.012, 0.328) compared to controls. No significant differences were found for BMI percentile. Narrative synthesis indicated limited evidence of effectiveness.

**Conclusions:**

Universal multicomponent healthy weight interventions for preschool children in high-income countries were associated with modest but meaningful improvements in zBMI, waist circumference, percentage body fat, and fat-free mass index. These findings suggest that early childhood interventions can be effective if delivered at scale and may serve as a key element in broader childhood obesity prevention strategies.

## Introduction

An estimated 39 million preschool children (aged 5 years or younger) live with overweight or obesity globally and face increased risk of various health conditions [1–4]. Once established in early childhood, obesity often tracks through adolescence and into adulthood [5]. The early years represent an important window of opportunity for prevention that could influence weight and health trajectories and reduce later obesity-related health risks [6].

There is evidence that healthy lifestyle behaviours, such as having a balanced diet and engaging in physical activity, are established early in life and are often sustained [7, 8]. Achieving a healthy weight before entering primary school is associated with reduced obesity-associated comorbidities [9], improved academic attainment [10], and greater emotional and social well-being [11].

In recent years, an increasing number of interventions focusing on pre-school children have been implemented in high-income countries (HICs) [12]. The National Institute of Clinical Excellent (NICE) [13] published evidence-based recommendations for targeted weight management interventions, emphasising the importance of multicomponent and multi-setting approaches. These structured, evidence-based guidelines provide a robust framework for developing effective interventions targeting childhood obesity prevention. While evidence suggests that targeted interventions (i.e. for individuals living with, or at risk of, overweight or obesity) can significantly lower body mass index (BMI) in pre-school children [14, 15], such interventions may not reach the broader population and do not act preventatively. Universal interventions (i.e. for individuals of any weight status) can have greater reach and potentially broader impact by addressing the environmental, social, and behavioural factors that contribute to obesity across all children, not just those already identified as high risk [16]. However, while prevention of obesity in children has been systematically assessed previously [12], evidence from universal interventions specifically targeting the early years has not yet been synthesised. Although universal interventions may produce small effects at the individual level, their broad reach can have a meaningful population health impact [17].

A United Kingdom (UK) based review [18] found that three of five universal multicomponent obesity interventions led to significant decreases in BMI *z*-scores (zBMI) [18]. Evidence from other HICs warrants exploration to support meaningful cross-country learning. The current study assesses interventions implemented in international settings that align with NICE guidelines and aims to synthesise the effects of universal healthy weight interventions for pre-school children on anthropometric outcomes, including zBMI, waist circumference, and weight status categories.

## Methods

The protocol for this systematic review and meta-analysis was pre-registered with PROSPERO (CRD42021290676) and was conducted following the Preferred Reporting Items for Systematic Reviews and Meta-Analyses [19] (PRISMA; see eTable 1, and Supplement 1). The Population, Intervention, Comparison, Outcomes and Study design [20] framework was utilised to formulate the eligibility criteria for studies included (See eTable 2, and Supplement 1). The data and scripts are available on GitHub (https://github.com/lk1373190/ey_obesity_meta).

### Data sources and search strategy

A systematic search for studies published since 2011 was conducted in 2021, followed by two updates in March 2023 and December 2024. Nine academic databases were systematically searched: CINAHL [EBSCO], Cochrane Library CENTRAL, Health Management Information Consortium [OVID], PubMed [NLM], PsycInfo [OVID], Scopus, Social Policy and Practice [OVID], Trials Register of Promoting Health Interventions, and Web of Science (SSCI, ESCI). Citation searches were also conducted. To identify and include data from unpublished trials, we conducted supplementary searches of clinical trial registries.

Search terms were based on a combination of descriptors including MeSH terms for exposure ((‘diet’ OR ‘food’ OR ‘beverages’ OR ‘food quality’ OR ‘food preferences’ OR ‘feeding behaviour*’ OR ‘exercise’ OR ‘sedentary behaviour’) AND (‘physical fitness’ OR ‘weight reduction programmes’ OR ‘health education’ OR ‘health promotion’ OR ‘primary prevention’ OR ‘pilot projects’ OR ‘feasibility studies’ OR ‘programme evaluation’)) and anthropometric measures (‘obesity’ OR ‘body mass index’ OR ‘body weight’ OR ‘body size’ OR ‘body composition’ OR ‘body weight changes’). Search terms are reported in full in the eTable 3, and Supplement 1.

### Eligibility criteria

The review included studies targeting preschool children (i.e. 5 years and under) in HICs, with no restrictions based on gender, ethnicity, socioeconomic status (SES), or other characteristics. Studies were eligible if they evaluated anthropometric outcomes, such as zBMI, BMI percentiles, adipose tissue measurements, or changes in weight classification. We included real-world intervention studies, i.e. randomised controlled trials (RCTs), cluster-RCTs, quasi-experimental and experimental studies with pre-post assessments. No restrictions on language.

To ensure the inclusion of robust and replicable interventions, we selected studies that adhered to NICE guidelines for weight management in children [13]. Therefore, all included studies featured multicomponent interventions incorporating diet, physical activity, and/or behavioural change elements. Interventions could be delivered across various settings, including homes, preschools, childcare facilities, healthcare environments, or through a combination of these approaches (multi-setting). Studies published from 2011 onwards were selected to ensure a contemporary evidence base, reflecting current interventions, policy and environmental contexts, as well as the use of more standardised and rigorous outcome measures in obesity research.

Studies were excluded if they: included children older than 5 years; based outside of high-income countries; were limited to prenatal/antenatal periods; lacked diet, physical activity and/or behavioural change components; did not measure anthropometric outcomes. We also excluded national policies or services, qualitative studies, reviews, dissertations, commentaries, editorials, conference proceedings, case studies, books, and opinion pieces.

### Study selection

Initially, 6779 studies were double-screened on title and abstract by two reviewers (MS and SM) using EPPI-Reviewer 6 software [21]. Discrepancies were resolved through discussion between the reviewers. Given the large volume of records identified in searches, we used the machine learning to support the screening process. An active learning approach was adopted, in which the system continuously updated the prioritisation of records, allowing the most likely relevant studies to be screened first. The algorithm was trained based on previous screening decisions. We tracked the number of included studies over time, using the point at which the inclusion rate levelled off as a stopping rule (suggesting few relevant studies remained). A classifier was then generated and applied to the unscreened records, assigning relevance scores (ranging from 0 to 100) to help verify exclusions. Reviewers independently screened all records with a score higher than 30. Full-text articles of all potentially eligible studies were reviewed by at least two authors (DD, MS, SM).

### Data extraction

Two authors (DD and LK) independently extracted data from all eligible studies, including information on study characteristics (primary author, publication year, country, study design), intervention or programme characteristics (intervention details), and key findings (changes pre- to post-intervention and impact of SES). See eAppendix 1. Supplementary methods in Supplement 1 for further information regarding the included outcome measures.

### Quality assessment

We used the following two quality assessment tools developed by the National Institutes of Health (NIH) [22]: the Quality Assessment of Controlled Intervention Studies, and the Quality Assessment Tool for Before-After (Pre-Post) Studies With No Control Group [22]. The certainty of evidence was evaluated using the Grading of Recommendation, Assessment, Development, and Evaluation (GRADE) framework [23]. Assessments were carried out independently by two authors (DD and LK), and discrepancies were jointly reconciled.

### Data analysis

For narrative synthesis, we explored study characteristics, intervention types, outcome measures, and the relationship between exposure and outcomes. Improvement was broadly defined as: decreases in BMI, BMI percentiles, zBMI, body weight measures (i.e. body weight (kg) and weight *z*-scores), fat mass index, percentage body fat, skinfold measures (i.e. triceps skinfold, bicep skinfold or subscapular skinfold), waist measures (i.e. waist circumference and waist-to-hip ratio); increases in fat-free mass index; changes in weight status categories.

Studies were included in meta-analyses (rationale for inclusion and exclusion from meta-analysis see eTable 4, and Supplement 1) if they were deemed sufficiently homogenous (i.e. used similar study designs [e.g. RCTs], outcomes [e.g. zBMI] and comparison groups [e.g. waitlist control]). To increase reliability in the estimates, at least three studies reporting the required data were required. For meta-analyses, baseline and follow-up means, along with their associated variances, were used. If standard deviations of the reported parameters were not available, we used the reported sample sizes and other measures (e.g. standard errors, confidence intervals) to calculate them or contacted the study authors for these data (see eTable 5 in Supplement 1 for calculations). The authors of five studies were contacted, and of these, additional data was provided by the authors of three studies (authors of two studies did not respond).

To account for statistical dependence among multiple effect sizes derived from the same study (e.g. multiple follow-up time points or intervention groups), we used multilevel random-effects meta-analysis. All models included a study-level random effect to capture between-study heterogeneity. In addition, models initially allowed for an effect-size-level random effect nested within study to capture within-study heterogeneity between effect sizes. The necessity of the additional effect-size-level variance component was evaluated separately for each outcome using likelihood ratio tests, Akaike Information Criterion and Bayesian Information Criterion. Where supported by the data, three-level models (including both study-level and effect-size–level random effects) were retained. Where not supported, a simpler two-level random-effects model including only the study-level random effect was used. This approach follows recommended practice to balance appropriate modelling of dependence with model parsimony [24, 25].

To evaluate publication bias, we created funnel plots to check for asymmetry in the distribution of estimates according to their precision. We conducted Egger’s test of heterogeneity [26] and examined the *I*^2^ statistic, which indicates the percentage of variability in effect estimates due to heterogeneity rather than sampling error. We classified heterogeneity as low (*I*^*2*^ ≤ 25%), moderate (*I*^*2*^ ≤ 50% and >25%) or high (*I*^*2*^ > 50%) [27]. To identify potential sources of heterogeneity in the association between interventions and outcomes, we ran moderator analyses according to intervention length and follow-up time. All analyses were conducted in R (version 4.2.3 [2023-03-15]) using the *metafor* [28] package (version 4.6-0).

## Results

**Fig. 1:**
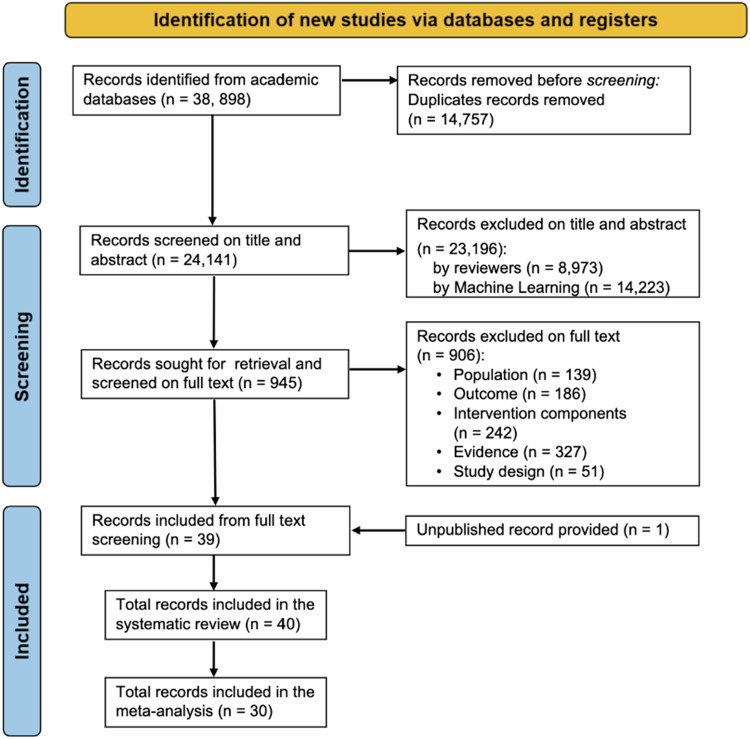
PRISMA flow diagram of the records identified, screened and included in the systematic review.

A total of 38,898 records were identified from academic database searches, of which 14,757 records were removed as duplicates. 24,141 records were screened on title and abstract, and 945 on full text. One unpublished record was provided directly by a study author. Forty studies were included in the narrative synthesis, of which 30 studies were included in meta-analyses (Fig. 1; see eTable 6, and Supplement 1 for a detailed descriptive table).

The 40 studies included 26,977 participants (baseline sample sizes ranged from 42 to 2658; mean = 674; eTable 7). Half of included studies were cluster RCTs [29–48] (*k* = 20; 50%), followed by RCTs [49–63] (*k* = 15; 37.5%), and experimental studies [64–68] (*k* = 5; 12.5%). The majority of samples were based in the United States of America (number of studies [*k*]=18; 45%), followed by Sweden (*k* = 5; 13%), Australia (*k* = 2; 5%), the UK (*k* = 2; 5%) and Germany (*k* = 2; 5%).

Of the 40 studies, 37 unique universal interventions were identified that aligned with the NICE guidelines for weight management in children. All interventions incorporated components targeting both nutrition and physical activity (*k* = 37; 100%). The majority addressed additional lifestyle factors such as sleep and sedentary behaviour (*k* = 31; 84%). Interventions were delivered across a range of settings: in person (*k* = 29; 78%); via digital applications (*k* = 3; 8%); telephone formats (*k* = 1; 3%); and multi-modal approaches (*k* = 4; 11%). The duration of interventions ranged from six to 39 months, with follow-up periods ranging from 6 to 66 months.

Twelve different anthropometric outcomes were reported: zBMI (*k* = 27 studies; 68%); weight status categories (*k* = 14; 35%), BMI (*k* = 14; 35%), and BMI percentiles (*k* = 11; 28%). Measures for weight were assessed in eight studies (20%) including body weight in kilograms (*k* = 6) and weight *z*-score (*k* = 2). Waist measures were reported in eight studies (20%), including waist circumference (*k* = 7) and waist-to-hip ratio (*k* = 1). Body composition indicators were less commonly reported, including skinfold measures (*k* = 5; 13%), percentage body fat (*k* = 3; 8%), fat-free mass index (*k* = 3; 8%) and fat mass index (*k* = 2; 5%). See eTable 7 in Supplement 1 for a descriptive summary of the participant characteristics and study features of the studies included. Meta-analyses were conducted for five anthropometric outcomes: zBMI, BMI percentiles, waist circumference, percentage body far and fat-free mass index.

### zBMI

**Fig. 2:**
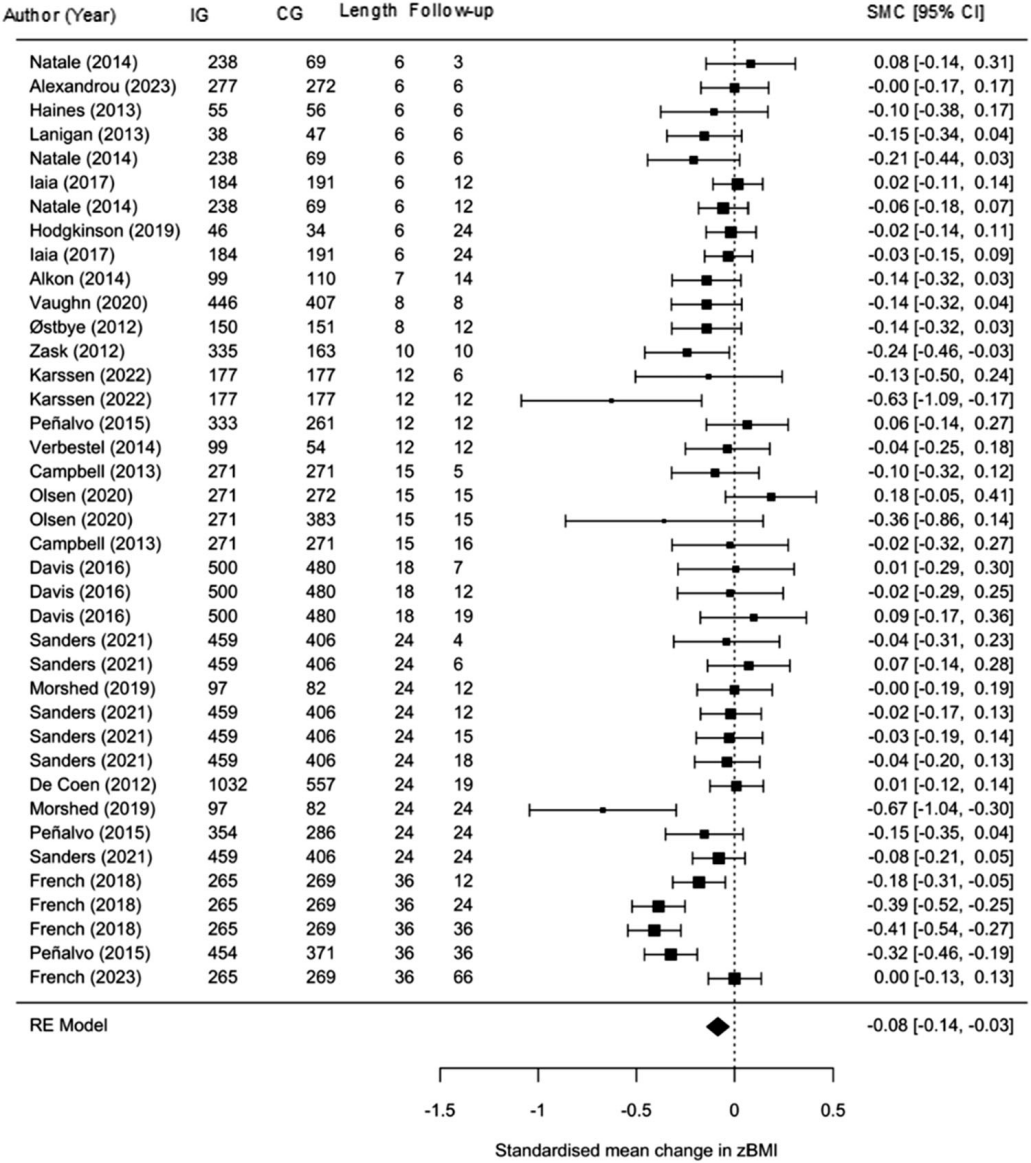
Forest plot of the effect of interventions on zBMI. IG = number of individuals in the intervention group; CG = number of individuals in the control group; length = length of intervention (months); follow-up = length of follow-up (months); SMC = standardised mean change; CI = confidence interval.

A total of 27 studies reported the effect of interventions on zBMI [29–32, 34–36, 39, 41, 42, 44, 45, 47–49, 52, 53, 55–59, 62, 65, 67, 68]. Of these, 21 studies (number of effects sizes [ES] = 39) were included in the meta-analysis. Meta-analysis suggested a small effect of interventions on zBMI (pooled *SMD* = −0.085; 95% CI = −0.140, −0.029; *n* = 10,849; Fig. 2 and eTable 8, Supplement 1), with interventions associated with a 0.085 unit decrease in zBMI. There was evidence of substantial heterogeneity (*I*^*2*^ = 59.32%). The remaining six studies could not be included in the meta-analysis as they did not have a waitlist or control group [36, 42, 47, 65, 67, 68]. The results reported in these studies were inconsistent with meta-analysis. Two studies reported a significant decrease in zBMI in the intervention group compared to control; the remaining four reported no difference between intervention and control.

### Waist measures

**Fig. 3:**
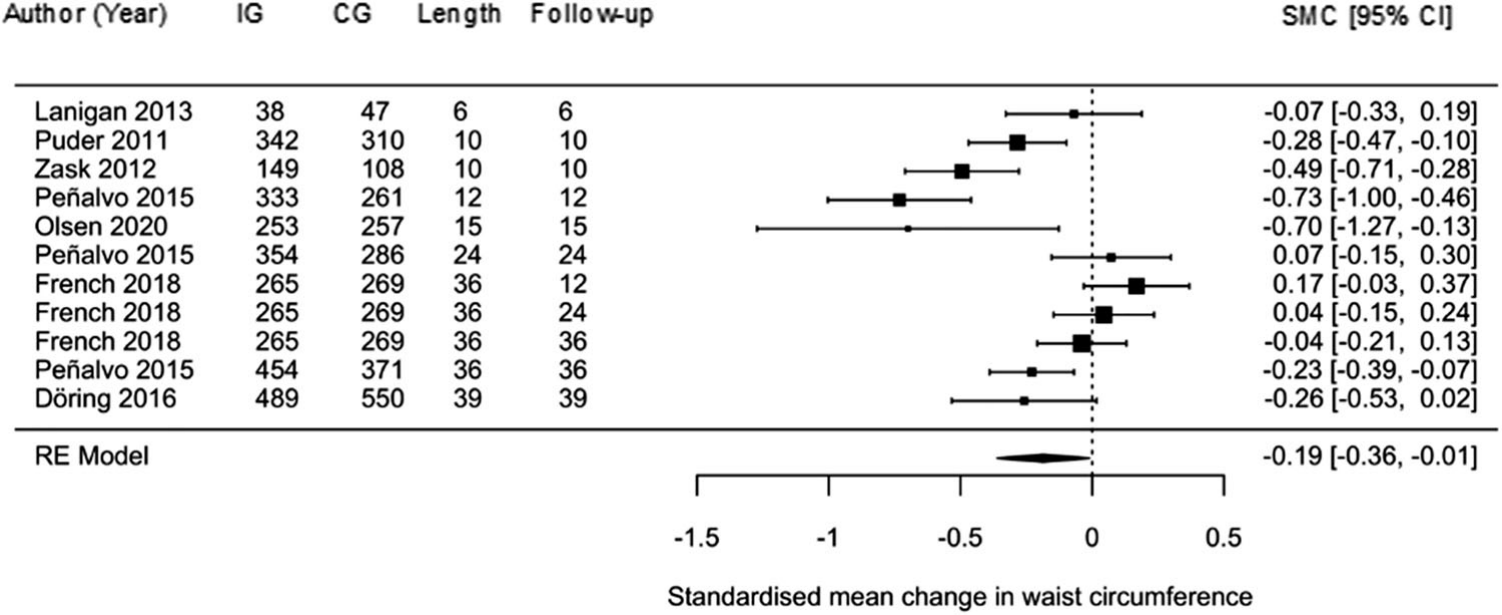
Forest plot of the effect of interventions on waist circumference. IG = number of individuals in the intervention group; CG = number of individuals in the control group; length = length of intervention (months); follow-up = length of follow-up (months); SMC = standardised mean change; CI = confidence interval.

Seven studies (ES = 11) measured waist circumference and were included in the meta-analysis [33, 39, 40, 48, 53, 57, 62]. Results showed a small effect of interventions on waist circumference (pooled *SMD* = −0.186; 95% CI = −0.361, −0.011; *n* = 3,902; Fig. 3 and eTable 8, Supplement 1), suggesting interventions were associated with a 0.186 cm decrease in waist circumference. There was evidence of substantial effect heterogeneity (*I*^*2*^ = 77.26%). One study, which was not included in the meta-analysis, measured waist-to-hip ratio, reported no significant differences between intervention groups [62].

### BMI percentiles

**Fig. 4:**
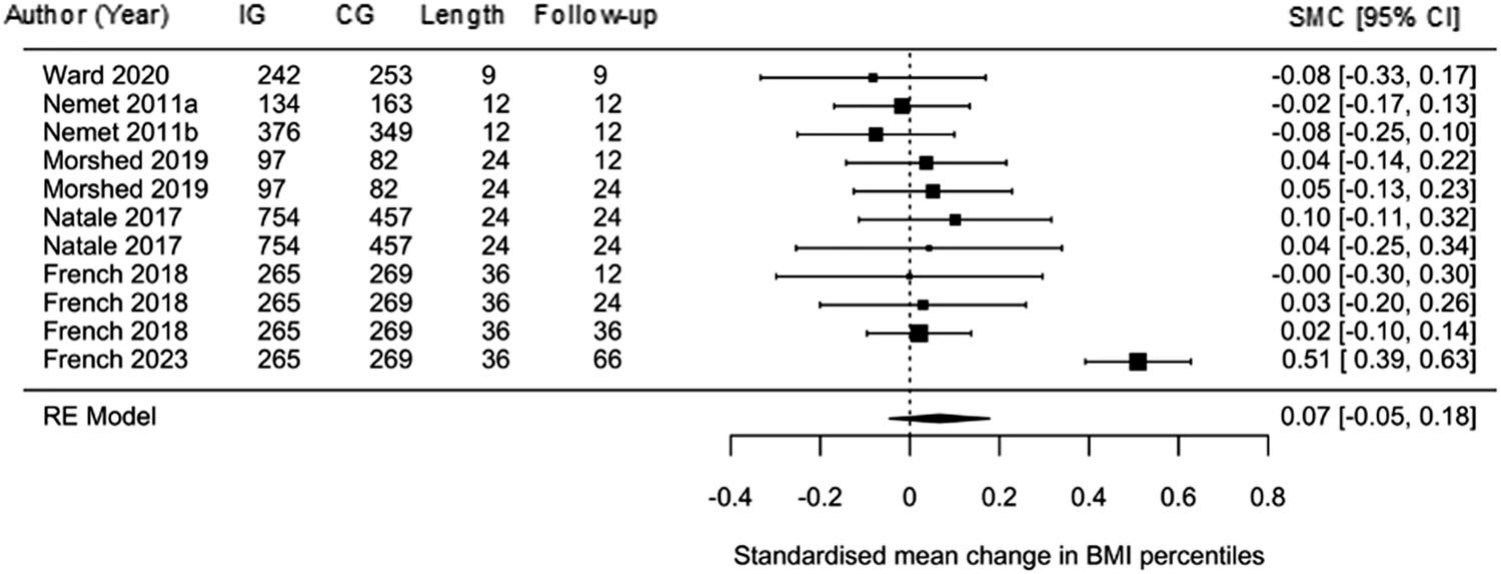
Forest plot of the effect of interventions on BMI percentiles. IG = number of individuals in the intervention group; CG = number of individuals in the control group; length = length of intervention (months); follow-up = length of follow-up (months); SMC = standardised mean change; CI = confidence interval.

11 studies measured BMI percentiles [37, 38, 42, 46, 47, 52, 53, 58, 60, 64, 65], of which seven studies (ES = 11) were included in a meta-analysis. Results suggested no effect of interventions on BMI percentiles (pooled *SMD* = 0.066; 95% CI = −0.045, 0.178; *n* = 3,975; Fig. 4 and eTable 8, Supplement 1). There was evidence of substantial heterogeneity (*I*^*2*^ = 75.60%). The four studies not included in the meta-analysis did not have a waitlist or control group [42, 47, 64, 65]. One study reported no significant difference, while three studies reported that BMI percentile significantly lowered among children in the intervention groups compared to controls.

### Percentage body fat

**Fig. 5:**
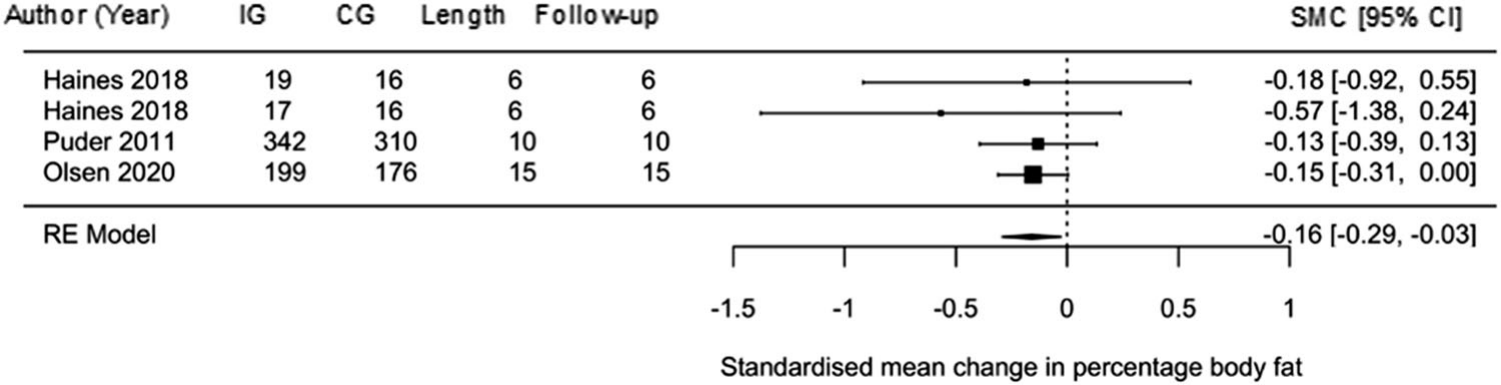
Forest plot of the effect of interventions on percentage body fat. IG = number of individuals in the intervention group; CG = number of individuals in the control group; length = length of intervention (months); follow-up = length of follow-up (months); SMC = standardised mean change; CI = confidence interval.

Three studies measured percentage body fat (ES = 4) and were included in a meta-analysis [40, 54, 66]. Results suggested an effect of interventions on percentage body fat (pooled *SMD* = −0.159; 95% CI = -0.290, −0.028; *n* = 1079; Fig. 5 and eTable 8, Supplement 1), interventions were associated with a 16% decrease in percentage body fat. There was no evidence of heterogeneity (*I*^*2*^ < 0.001). However, given the small number of studies, estimates of heterogeneity are imprecise and should be interpreted cautiously.

### Fat-free mass index

**Fig. 6:**
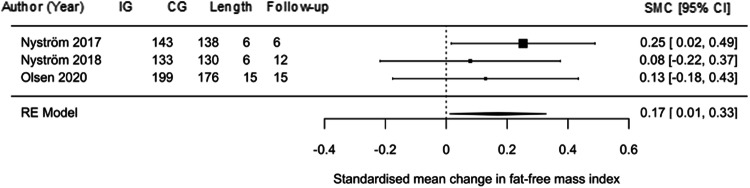
Forest plot of the effect of interventions on fat-free mass index. IG = number of individuals in the intervention group; CG = number of individuals in the control group; length = length of intervention (months); follow-up = length of follow-up (months); SMC = standardised mean change; CI = confidence interval.

Three studies measured fat-free mass index (ES = 3) and were included in a meta-analysis [50, 61, 62]. Results suggested an effect of interventions on fat-free mass index (pooled *SMD* = 0.170; 95% CI = 0.012, 0.328; *n* = 919; Fig. 6 and eTable 8 in Supplement 1). There was no effect heterogeneity (*I*^*2*^ < 0.001), although the small number of effect sizes limits interpretability.

### Fat mass index

Two studies measured fat mass index and reported no significant changes after intervention [50, 61].

### BMI

Fourteen studies assessed changes in BMI post-intervention [33, 35, 37–40, 44, 46, 47, 51–53, 55, 57]. Results suggested a limited effect of intervention on BMI, with 12 studies reporting no significant difference in BMI and two studies reporting significant BMI changes in the intervention group.

### Weight status categories

Fourteen studies assessed changes in weight status, before and after an intervention [29, 33, 36, 39–43, 48, 51, 52, 58, 65, 66], including the prevalence of overweight, obesity, and the combined prevalence of both. Five studies examined changes in the combined prevalence of overweight and obesity: one reported a statistically significant reduction, and four found no significant difference. Six studies assessed changes in the prevalence of obesity: two reported significant reductions, and four reported no significant change. Nine studies evaluated no changes in the prevalence of overweight.

### Body weight measures

Eight studies reported body weight outcomes, of which six measured body weight and reported no significant difference post-intervention [37, 38, 50, 53, 57, 61]. There were an insufficient number of studies (*k* = 2) to conduct a separate meta-analysis on weight *z*-scores [47, 59]. One study reported no significant differences in weight *z*-scores [59]. The other study with two intervention groups (one in a childcare centre only and the another set in a childcare centre with home-based components); both groups showed significant reductions in weight *z*-scores compared to controls [47].

### Skinfolds measures

Skinfold measures were reported in five studies [39, 40, 53, 57, 62]. The sum of four skinfolds (in mm) was reported in three studies, the triceps skinfold (in cm) in one study, and both subscapular and triceps skinfold *z*-scores were reported in one study. One study reported a significant reduction in the sum of four skinfolds compared to control. The intervention effect corresponded to a 10% decrease from the baseline mean value (SMD = −2.78; 95% CI = −4.35, −1.2) [40].

### Publication bias

The results for zBMI (*Egger* = 101.40, *p* < 0.001; eFigure 1, and Supplement 1), waist circumference (*Egger* = 53.63, *p* < 0.001; eFigure 2, and Supplement 1) and BMI percentiles (*Egger* = 57.95, *p* < 0.001; eFigure 3, and Supplement 1), suggested evidence of potential publication bias in the included studies. The results for percentage body fat (*Egger* = 1.04, *p* = 0.308; eFigure 4, and Supplement 1) fat-free mass index (*Egger* = 0.90, *p* = 0.342; eFigure 5, and Supplement 1), suggested no evidence of publication bias.

### Sensitivity analyses

Moderator analyses indicated that intervention length did not influence the effect of interventions on any outcome, suggesting that the observed pooled effects were robust to variation in intervention duration (eTable 9, and Supplement 1).

Of the 40 studies included in the review, five examined whether SES influenced the effect of the intervention (eTable 10, and Supplement 1). Two studies reported no impact of the intervention when parental education was used as a moderator [49, 51]. In contrast, two studies found the intervention to be more effective among parents with lower educational levels [56] or only significant within low-SES communities [32]. Finally, one study observed a significant positive effect among children whose mothers had a medium/high level of education compared to those with low education [35]. An additional five studies adjusted for SES in their analyses but did not examine its impact on the effect of interventions [29, 30, 61, 62, 66].

### Risk of bias and quality assessment

The NIH Quality Assessment Tool for Controlled Intervention Studies was applied to 34 included studies. Of these, 17 (50%) were rated as good quality, 14 (41%) as fair, and 3 (9%) as poor (see eFigure 6, and Supplement 1). The remaining six studies were assessed using the NIH Quality Assessment Tool for Before-After (Pre-Post) Studies With No Control Group. Among these, four studies (66.7%) were rated as good quality, and two studies (33.3%) were rated as fair (see eFigure 7, and Supplement 1). A common risk was the lack of blinding of participants, intervention providers, and outcome assessors, and insufficient reporting on adherence to intervention.

The GRADE [23] framework certainty of the evidence is presented in eTable 11, and Supplement 1. For studies included in meta-analysis, the certainty of evidence was moderate for studies reporting zBMI, waist circumference and BMI percentile, and high for studies reporting fat mass index and fat-free mass index. For studies narratively synthesised, the certainty of evidence was high for BMI, moderate for skinfold and weight measures, and low for weight status categories and percentage body fat.

## Discussion

This review assessed the effectiveness of universal healthy weight interventions for preschool children in HICs. A range of anthropometric outcomes were synthesised in meta-analyses and narratively. The meta-analytic findings suggested that interventions were associated with decreases in zBMI and waist circumference and increases in fat-free mass index; no differences were observed in BMI percentiles and fat-mass index. Narrative synthesis showed limited evidence of intervention effectiveness, with most studies reporting no significant difference in weight status, BMI, body weight, skinfold measures, or body fat percentage. These findings suggest that multicomponent interventions can support healthy growth trajectories in preschool children and represent a valuable component in childhood obesity prevention strategies. Our pooled effect size for zBMI (−0.08) is similar to previous estimates (−0.07) [14], demonstrating modest but meaningful effects for children of any weight status. Although this magnitude of change is small at the individual level, evidence suggests that even minimal reductions or stabilisation of BMIz in early childhood are associated with favourable cardiometabolic outcomes and may alter long-term obesity trajectories [69, 70]. Modelling studies support the cost-effectiveness of substantial reductions in long-term healthcare costs [71, 72].

Effective interventions shared several key characteristics. Most were implemented in childcare settings while engaging parents through workshops, educational materials and home-based activities. Two successful interventions (The Programme SI![Spain] [39] and NET-Works[USA] [52, 53]) were large, well-evaluated, spanned multiple settings (e.g. homes, childcare and healthcare centres), incorporated creative, engaging and playful approaches, engaged multiple stakeholders (e.g. children, parents and carers/teachers) and addressed broader lifestyle factors, including media use and sleep patterns.

The effect sizes reported in the current review were pooled from controlled research trials and the scalability and ‘real-world’ effectiveness is unknown, representing a challenge for policymakers and public health leads [73]. Implementation frameworks, such as the PIET-T model (P: population, I: intervention, E: environment, T: transfer, -T: transferability), can facilitate careful scaling by systematically assessing contextual factors and the likelihood of intervention transferability [74].

Evidence demonstrates that intervention engagement is unequal across socioeconomic groups [75], highlighting the importance of identifying and addressing the barriers faced by disadvantaged communities. Research is needed to understand the specific challenges faced by marginalised groups to co-develop solutions. Improving intervention equity may require enhancing the affordability of healthy food, access to healthcare services for disadvantaged families, or improving structural provisions such as facilities and green space [76]. Future interventions should incorporate equity considerations from design through implementation to ensure equitable benefits of interventions.

The long-term effectiveness of early childhood interventions remains unclear; only 12 studies in the current review included a follow-up post-intervention. However, follow-up duration was not found to influence intervention effectiveness, suggesting that, for preventative interventions the content and delivery of interventions may be key to promoting healthier developmental trajectories.

### Strengths and limitations

This review considered traditional anthropometric outcomes, including BMI and zBMI. While these measures are widely used and offer practical advantages, they may not accurately measure adiposity in young children, especially for certain ethnic groups. Relying on zBMI to indicate effectiveness may restrict the assessment of interventions and overlook changes in body composition. Our review included a range of anthropometric outcomes, including waist circumference, fat mass index, and fat-free mass index, which provide a more accurate measure of adiposity and better reflect changes in fat distribution and lean tissue. Including these measures provides a better understanding of how universal interventions influence growth trajectories and provides evidence to support comprehensive evaluation of childhood obesity interventions.

While many individual studies reported no statistically significant effect on BMIz, and others showed benefits only at certain time points, meta-analysis provides a robust approach to synthesising evidence across studies. This enhances statistical power, identifies overall patterns, and informs public health policy, even when single studies yield mixed or null effects [77]. This review employed rigorous methodology, including machine learning-assisted screening and comprehensive database searches. We focused exclusively on objectively measured anthropometric outcomes to minimise bias, though this approach may have excluded interventions with positive behavioural impacts with unmeasurable anthropometric change.

Several limitations warrant consideration. Our machine learning approach to screening may have missed eligible studies. We believe this is unlikely given our use of a classifier model applied to unscreened items. The decision to exclude studies published before 2011 was informed by a previous meta-analysis [14], which found very few relevant studies prior to 2011. Studies published before 2011 are likely to have less relevance to contemporary contexts and are unlikely to substantially affect findings [78]. We considered objectively recorded anthropometric outcomes and did not consider interventions that target behaviours that influence energy balance. A limitation of the literature that effects the overall quality of evidence is that many studies had high risk of bias in at least one of the assessed domains, commonly in the blinding of participants or researchers. Additionally, only five out of 40 studies explicitly examined whether SES moderated intervention effects, and an additional 5 adjusted for it. This limited evidence highlights the need for future research to systematically assess SES as a potential moderator, using consistent measures and reporting standards to better understand equity impacts.

## Conclusions

Universal multicomponent healthy weight interventions targeting preschool children in HICs demonstrate modest but potentially meaningful effects on anthropometric outcomes, particularly zBMI and waist circumference. Although fat-free mass index was examined in only three studies, the significant positive effects observed warrant further investigation, as increases in lean body mass represent an important indicator of healthy growth and development. Translating research findings into successful real-world implementation requires systematic attention to equity considerations, long-term sustainability mechanisms, and evidence-based scaling frameworks. Future research priorities should include further examining the effect of interventions on fat-free mass outcomes, extended follow-up periods to assess intervention durability and higher-quality study designs with improved methodological rigour. The findings underscore the importance of greater investment in prevention as a key strategy for promoting healthy weight in children.

## Supplementary information

Supplement 1

## References

[1] World Health Organization. Obesity and overweight 2025. https://www.who.int/news-room/fact-sheets/detail/obesity-and-overweight.

[2] Abbasi A, Juszczyk D, van Jaarsveld CHM, Gulliford MC. Body mass index and incident type 1 and type 2 diabetes in children and young adults: a retrospective cohort study. J Endocr Soc. 2017;1:524–37. 29264507 10.1210/js.2017-00044 PMC5686575

[3] Lindberg L, Hagman E, Danielsson P, Marcus C, Persson M. Anxiety and depression in children and adolescents with obesity: a nationwide study in Sweden. BMC Med. 2020;18:30. 32079538 10.1186/s12916-020-1498-z PMC7033939

[4] Umer A, Kelley GA, Cottrell LE, Giacobbi P Jr, Innes KE, Lilly CL. Childhood obesity and adult cardiovascular disease risk factors: a systematic review with meta-analysis. BMC Public Health. 2017;17:683. 28851330 10.1186/s12889-017-4691-z PMC5575877

[5] Ward ZJ, Long MW, Resch SC, Giles CM, Cradock AL, Gortmaker SL. Simulation of growth trajectories of childhood obesity into adulthood. N Engl J Med. 2017;377:2145–53. 29171811 10.1056/NEJMoa1703860 PMC9036858

[6] Natale RA, Messiah SE, Asfour L, Uhlhorn SB, Delamater A, Arheart KL. Role modeling as an early childhood obesity prevention strategy: effect of parents and teachers on preschool children’s healthy lifestyle habits. J Dev Behav Pediatr. 2014;35:378–87. 25007060 10.1097/DBP.0000000000000074

[7] Dos Santos CS, Picoito J, Nunes C, Loureiro I. Early individual and family predictors of weight trajectories from early childhood to adolescence: results from the millennium cohort study. Front Pediatr. 2020;8:417. 32850533 10.3389/fped.2020.00417 PMC7431491

[8] Geserick M, Vogel M, Gausche R, Lipek T, Spielau U, Keller E, et al. Acceleration of BMI in early childhood and risk of sustained obesity. N Engl J Med. 2018;379:1303–12. 30281992 10.1056/NEJMoa1803527

[9] Wijga AH, Scholtens S, Bemelmans WJE, de Jongste JC, Kerkhof M, Schipper M, et al. Comorbidities of obesity in school children: a cross-sectional study in the PIAMA birth cohort. BMC Public Health. 2010;10:184. 20380692 10.1186/1471-2458-10-184 PMC2858121

[10] Watson A, D’Souza NJ, Timperio A, Cliff DP, Okely AD, Hesketh KD. Longitudinal associations between weight status and academic achievement in primary school children. Pediatr Obes. 2023;18:e12975. 36128712 10.1111/ijpo.12975 PMC10078458

[11] Pryor L, Brendgen M, Boivin M, Dubois L, Japel C, Falissard B, et al. Overweight during childhood and internalizing symptoms in early adolescence: the mediating role of peer victimization and the desire to be thinner. J Affect Disord. 2016;202:203–9. 27267292 10.1016/j.jad.2016.05.022

[12] Brown T, Moore TH, Hooper L, Gao Y, Zayegh A, Ijaz S, et al. Interventions for preventing obesity in children. Cochrane Database Syst Rev. 2019;7:Cd001871. 31332776 10.1002/14651858.CD001871.pub4 PMC6646867

[13] NICE Evidence Reviews Collection. Evidence review for effectiveness and acceptability of weight management interventions in children and young people living with overweight and obesity: Overweight and obesity management: preventing, assessing and managing overweight and obesity: Evidence review G. London: National Institute for Health and Care Excellence (NICE) Copyright © NICE 2025; 2025. 40073177

[14] Colquitt JL, Loveman E, O’Malley C, Azevedo LB, Mead E, Al-Khudairy L, et al. Diet, physical activity, and behavioural interventions for the treatment of overweight or obesity in preschool children up to the age of 6 years. Cochrane Database Syst Rev. 2016;3:Cd012105. 26961576 10.1002/14651858.CD012105 PMC6669248

[15] Nordlund S, McPhee PG, Gabarin R, Deacon C, Mbuagbaw L, Morrison KM. Effect of obesity treatment interventions in preschool children aged 2-6 years: a systematic review and meta-analysis. Br Med J Open. 2022;12:e053523. 10.1136/bmjopen-2021-053523 PMC8984001 35383062

[16] Kumanyika SK, Obarzanek E, Stettler N, Bell R, Field AE, Fortmann SP, et al. Population-based prevention of obesity. Circulation. 2008;118:428–64. 18591433 10.1161/CIRCULATIONAHA.108.189702

[17] Rose G. Sick individuals and sick populations. Int J Epidemiol. 1985;14:32–8. 3872850 10.1093/ije/14.1.32

[18] Michalopoulou S, Sifaki M, Packer J, Lanigan J, Stansfield C, Viner RM, et al. Assessing the impact of obesity interventions in the early years: a systematic review of UK-based studies. Br Med J Open. 2024;14:e076479. 10.1136/bmjopen-2023-076479 PMC11097867 38740507

[19] Shamseer L, Moher D, Clarke M, Ghersi D, Liberati A, Petticrew M, et al. Preferred reporting items for systematic review and meta-analysis protocols (PRISMA-P) 2015: elaboration and explanation. Br Med J. 2015;349:g7647. 10.1136/bmj.g7647 25555855

[20] Frandsen TF, Bruun Nielsen MF, Lindhardt CL, Eriksen MB. Using the full PICO model as a search tool for systematic reviews resulted in lower recall for some PICO elements. J Clin Epidemiol. 2020;127:69–75. 32679315 10.1016/j.jclinepi.2020.07.005

[21] Thomas J, Graziosi, S, Brunton, J, Ghouze, Z, O’Driscoll, P, & Bond, M et al. EPPI-Reviewer: advanced software for systematic reviews, maps and evidence synthesis. EPPI Centre, UCL Social Research Institute, University College London; 2023.

[22] National Institutes of Health (NIH). Study Quality Assessment Tools 2013. https://www.nhlbi.nih.gov/health-topics/study-quality-assessment-tools.

[23] Foroutan F, Guyatt G, Zuk V, Vandvik PO, Alba AC, Mustafa R, et al. GRADE Guidelines 28: use of GRADE for the assessment of evidence about prognostic factors: rating certainty in identification of groups of patients with different absolute risks. J Clin Epidemiol. 2020;121:62–70. 31982539 10.1016/j.jclinepi.2019.12.023

[24] Assink M, Wibbelink CJM. Fitting three-level meta-analytic models in R: a step-by-step tutorial. Quant Methods Psychol. 2016;12:154–74.

[25] Cheung MW. A guide to conducting a meta-analysis with non-independent effect sizes. Neuropsychol Rev. 2019;29:387–96. 31446547 10.1007/s11065-019-09415-6 PMC6892772

[26] Rodgers MA, Pustejovsky JE. Evaluating meta-analytic methods to detect selective reporting in the presence of dependent effect sizes. Psychol Methods. 2021;26:141–60. 32673040 10.1037/met0000300

[27] Higgins JP, Thompson SG. Quantifying heterogeneity in a meta-analysis. Stat Med. 2002;21:1539–58. 12111919 10.1002/sim.1186

[28] Viechtbauer W. Conducting meta-analyses in R with the metafor package. J Stat Softw. 2010;36:1–48.

[29] Alkon A, Crowley AA, Neelon SEB, Hill S, Pan Y, Nguyen V, et al. Nutrition and physical activity randomized control trial in child care centers improves knowledge, policies, and children’s body mass index. BMC Public Health. 2014;14:215. 24580983 10.1186/1471-2458-14-215 PMC3945995

[30] Campbell KJ, Lioret S, McNaughton SA, Crawford DA, Salmon J, Ball K, et al. A parent-focused intervention to reduce infant obesity risk behaviors: a randomized trial. Pediatrics. 2013;131:652–60. 23460688 10.1542/peds.2012-2576

[31] Davis SM, Myers OB, Cruz TH, Morshed AB, Canaca GF, Keane PC, et al. CHILE: outcomes of a group randomized controlled trial of an intervention to prevent obesity in preschool Hispanic and American Indian children. Prev Med. 2016;89:162–8. 27222162 10.1016/j.ypmed.2016.05.018 PMC4969221

[32] De Coen V, De Bourdeaudhuij I, Vereecken C, Verbestel V, Haerens L, Huybrechts I, et al. Effects of a 2-year healthy eating and physical activity intervention for 3-6-year-olds in communities of high and low socio-economic status: the POP (Prevention of Overweight among Pre-school and school children) project. Public Health Nutr. 2012;15:1737–45. 22397833 10.1017/S1368980012000687

[33] Döring N, Ghaderi A, Bohman B, Heitmann BL, Larsson C, Berglind D, et al. Motivational interviewing to prevent childhood obesity: a cluster RCT. Pediatrics. 2016;137:e20153104. 27244793 10.1542/peds.2015-3104

[34] Hodgkinson A, Abbott J, Hurley MA, Lowe N, Qualter P. An educational intervention to prevent overweight in pre-school years: a cluster randomised trial with a focus on disadvantaged families. BMC Public Health. 2019;19:1430. 31675942 10.1186/s12889-019-7595-2 PMC6824038

[35] Iaia M, Pasini M, Burnazzi A, Vitali P, Allara E, Farneti M. An educational intervention to promote healthy lifestyles in preschool children: a cluster-RCT. Int J Obes. 2017;41:582–90. 10.1038/ijo.2016.239 28028319

[36] Lumeng JC, Miller AL, Horodynski MA, Brophy-Herb HE, Contreras D, Lee H, et al. Improving self-regulation for obesity prevention in head start: a randomized controlled trial. Pediatrics. 2017;139:e20162047. 28557722 10.1542/peds.2016-2047

[37] Nemet D, Geva D, Eliakim A. Health Promotion Intervention in Low Socioeconomic Kindergarten Children. J Pediatr. 2011;158:796–801.e1. 21244862 10.1016/j.jpeds.2010.10.040

[38] Nemet D, Geva D, Pantanowitz M, Igbaria N, Meckel Y, Eliakim A. Health promotion intervention in Arab-Israeli kindergarten children. J Pediatr Endocrinol Metab. 2011;24:1001–7. 22308855 10.1515/jpem.2011.387

[39] Peñalvo JL, Santos-Beneit G, Sotos-Prieto M, Bodega P, Oliva B, Orrit X, et al. The SI! program for cardiovascular health promotion in early childhood: a cluster-randomized trial. J Am Coll Cardiol. 2015;66:1525–34. 26429075 10.1016/j.jacc.2015.08.014

[40] Puder JJ, Marques-Vidal P, Schindler C, Zahner L, Niederer I, Bürgi F, et al. Effect of multidimensional lifestyle intervention on fitness and adiposity in predominantly migrant preschool children (Ballabeina): cluster randomised controlled trial. Br Med J. 2011;343:d6195. 21998346 10.1136/bmj.d6195 PMC3192456

[41] Sanders LM, Perrin EM, Yin HS, Delamater AM, Flower KB, Bian A, et al. A Health-literacy intervention for early childhood obesity prevention: a cluster-randomized controlled trial. Pediatrics. 2021;147:e2020049866. 33911032 10.1542/peds.2020-049866 PMC8086006

[42] Stookey JD, Evans J, Chan C, Tao-Lew L, Arana T, Arthur S. Healthy apple program to support child care centers to alter nutrition and physical activity practices and improve child weight: a cluster randomized trial. BMC Public Health. 2017;17:965. 29320996 10.1186/s12889-017-4951-y PMC6389251

[43] Strauß A, Herbert B, Mitschek C, Duvinage K, Koletzko B. TigerKids. Bundesgesundheitsblatt - Gesundheitsforschung - Gesundheitsschutz. 2011;54:322–9. 10.1007/s00103-010-1225-6 21347765

[44] Vaughn AE, Hennink-Kaminski H, Moore R, Burney R, Chittams JL, Parker P, et al. Evaluating a child care-based social marketing approach for improving children’s diet and physical activity: results from the Healthy Me, Healthy We cluster-randomized controlled trial. Transl Behav Med. 2021;11:775–84. 33231679 10.1093/tbm/ibaa113 PMC8033596

[45] Verbestel V, De Coen V, Van Winckel M, Huybrechts I, Maes L, De Bourdeaudhuij I. Prevention of overweight in children younger than 2 years old: a pilot cluster-randomized controlled trial. Public Health Nutr. 2013;17:1384–92. 23701835 10.1017/S1368980013001353 PMC10282209

[46] Ward DS, Vaughn AE, Burney RV, Hales D, Benjamin-Neelon SE, Tovar A, et al. Keys to healthy family child care homes: results from a cluster randomized trial. Prev Med. 2020;132:105974. 31899253 10.1016/j.ypmed.2019.105974 PMC8091030

[47] Yin Z, Liang Y, Howard JT, Errisuriz V, Estrada VM, Martinez C, et al. Míranos! a comprehensive preschool obesity prevention programme in low-income Latino children: 1-year results of a clustered randomised controlled trial. Public health Nutr. 2022;26:476–87. 36357340 10.1017/S1368980022002439 PMC10172390

[48] Zask A, Kaye Adams J, Owen Brooks L, Frances Hughes D. Tooty Fruity Vegie: an obesity prevention intervention evaluation in Australian preschools. Health Promot J Aust. 2012;23:10–5. 10.1071/he12010 22730932

[49] Alexandrou C, Henriksson H, Henström M, Henriksson P, Delisle Nyström C, Bendtsen M, et al. Effectiveness of a Smartphone App (MINISTOP 2.0) integrated in primary child health care to promote healthy diet and physical activity behaviors and prevent obesity in preschool-aged children: randomized controlled trial. Int J Behav Nutr Phys Act. 2023;20:22. 36810069 10.1186/s12966-023-01405-5 PMC9942425

[50] Delisle Nyström C, Sandin S, Henriksson P, Henriksson H, Maddison R, Löf M. A 12-month follow-up of a mobile-based (mHealth) obesity prevention intervention in pre-school children: the MINISTOP randomized controlled trial. BMC Public Health. 2018;18:658. 29793467 10.1186/s12889-018-5569-4 PMC5968487

[51] Enö Persson J, Bohman B, Tynelius P, Rasmussen F, Ghaderi A. Prevention of childhood obesity in child health services: follow-up of the PRIMROSE trial. Child Obes. 2018;14:99–105. 29232526 10.1089/chi.2017.0117

[52] French SA, Kunin-Batson AS, Sherwood NE, Berge JM, Shanley R. NET-Works paediatric obesity prevention trial: 66month outcomes. Pediatr Obes. 2023;18:e13055. 37171137 10.1111/ijpo.13055 PMC10462385

[53] French SA, Sherwood NE, Veblen-Mortenson S, Crain AL, JaKa MM, Mitchell NR, et al. Multicomponent obesity prevention intervention in low-income preschoolers: primary and subgroup analyses of the NET-works randomized clinical trial, 2012-2017. Am J Public Health. 2018;108:1695–706. 30403521 10.2105/AJPH.2018.304696 PMC6236759

[54] Haines J, Douglas S, Mirotta JA, O’Kane C, Breau R, Walton K, et al. Guelph Family Health Study: pilot study of a home-based obesity prevention intervention. Can J Public Health. 2018;109:549–60. 29981086 10.17269/s41997-018-0072-3 PMC6964565

[55] Haines J, McDonald J, O’Brien A, Sherry B, Bottino CJ, Schmidt ME, et al. Healthy Habits, Happy Homes: randomized trial to improve household routines for obesity prevention among preschool-aged children. JAMA Pediatr. 2013;167:1072–9. 24019074 10.1001/jamapediatrics.2013.2356

[56] Karssen LT, Larsen JK, Burk WJ, Kremers SPJ, Hermans RCJ, Ruiter ELM, et al. Process and effect evaluation of the app-based parenting program Samen Happie! on infant zBMI: a randomized controlled trial. Front Public Health. 2022;10:1012431. 36620259 10.3389/fpubh.2022.1012431 PMC9822729

[57] Lanigan J, Collins S, Birbara T, Kokoreli M, Singhal A. The TrimTots programme for prevention and treatment of obesity in preschool children: evidence from two randomised controlled trials. The Lancet. 2013;382:S58.

[58] Morshed AB, Tabak RG, Schwarz CD, Haire-Joshu D. The impact of a healthy weight intervention embedded in a home-visiting program on children’s weight and mothers’ feeding practices. J Nutr Educ Behav. 2019;51:237–44. 30385250 10.1016/j.jneb.2018.09.001 PMC6392451

[59] Natale RA, Lopez-Mitnik G, Uhlhorn SB, Asfour L, Messiah SE. Effect of a child care center-based obesity prevention program on body mass index and nutrition practices among preschool-aged children. Health Promot Pract. 2014;15:695–705. 24662896 10.1177/1524839914523429

[60] Natale RA, Messiah SE, Asfour LS, Uhlhorn SB, Englebert NE, Arheart KL. Obesity prevention program in childcare centers: two-year follow-up. Am J Health Promot. 2017;31:502–10. 27630110 10.1177/0890117116661156

[61] Nyström CD, Sandin S, Henriksson P, Henriksson H, Trolle-Lagerros Y, Larsson C, et al. Mobile-based intervention intended to stop obesity in preschool-aged children: the MINISTOP randomized controlled trial1,2. Am J Clin Nutr. 2017;105:1327–35. 28446496 10.3945/ajcn.116.150995

[62] Olsen NJ, Ängquist L, Frederiksen P, Lykke Mortensen E, Heitmann BL. Primary prevention of fat and weight gain among obesity susceptible healthy weight preschool children. Main results from the “Healthy Start” randomized controlled intervention. Pediatr Obes. 2021;16:e12736. 33021348 10.1111/ijpo.12736

[63] Østbye T, Krause KM, Stroo M, Lovelady CA, Evenson KR, Peterson BL, et al. Parent-focused change to prevent obesity in preschoolers: Results from the KAN-DO study. Prev Med. 2012;55:188–95. 22705016 10.1016/j.ypmed.2012.06.005 PMC3439558

[64] Cloutier MM, Wiley J, Huedo-Medina T, Ohannessian CM, Grant A, Hernandez D, et al. Outcomes from a pediatric primary care weight management program: steps to growing up healthy. J Pediatr. 2015;167:372–7.e1. 26073106 10.1016/j.jpeds.2015.05.028

[65] Sharma SV, Vandewater E, Chuang R-J, Byrd-Williams C, Kelder S, Butte N, et al. Impact of the coordinated approach to child health early childhood program for obesity prevention among preschool children: the texas childhood obesity research demonstration study. Child Obes. 2018;15:1–13. 30226991 10.1089/chi.2018.0010

[66] Steenbock B, Buck C, Zeeb H, Rach S, Pischke CR. Impact of the intervention program “JolinchenKids – fit and healthy in daycare” on energy balance related-behaviors: results of a cluster controlled trial. BMC Pediatr. 2019;19:432. 31722702 10.1186/s12887-019-1817-8 PMC6852984

[67] van de Kolk I, Gerards SMPL, Harms LSE, Kremers SPJ, Gubbels JS. The effects of a comprehensive, integrated obesity prevention intervention approach (SuperFIT) on children’s physical activity, sedentary behavior, and BMI Z-score. Int J Environ Res Public Health. 2019;16:5016. 31835477 10.3390/ijerph16245016 PMC6950277

[68] Woo Baidal JA, Nelson CC, Perkins M, Colchamiro R, Leung-Strle P, Kwass J-A, et al. Childhood obesity prevention in the Women, Infants, and Children Program: Outcomes of the MA-CORD study. Obesity. 2017;25:1167–74. 28653498 10.1002/oby.21865 PMC5600510

[69] Kolsgaard MLP, Joner G, Brunborg C, Anderssen SA, Tonstad S, Andersen LF. Reduction in BMI z-score and improvement in cardiometabolic risk factors in obese children and adolescents. The Oslo Adiposity Intervention Study - a hospital/public health nurse combined treatment. BMC Pediatr. 2011;11:47. 21619652 10.1186/1471-2431-11-47 PMC3121603

[70] Mears R, Salway R, Sharp D, Shield JPH, Jago R. A longitudinal study investigating change in BMI z-score in primary school-aged children and the association of child BMI z-score with parent BMI. BMC Public Health. 2020;20:1902. 33302899 10.1186/s12889-020-10001-2 PMC7731748

[71] Welsh BC, Sullivan CJ, Olds DL. When early crime prevention goes to scale: a new look at the evidence. Prev Sci. 2010;11:115–25. 19936922 10.1007/s11121-009-0159-4

[72] Brown V, Ananthapavan J, Sonntag D, Tan EJ, Hayes A, Moodie M. The potential for long-term cost-effectiveness of obesity prevention interventions in the early years of life. Pediatr Obes. 2019;14:e12517. 30816024 10.1111/ijpo.12517

[73] McCrabb S, Lane C, Hall A, Milat A, Bauman A, Sutherland R, et al. Scaling-up evidence-based obesity interventions: a systematic review assessing intervention adaptations and effectiveness and quantifying the scale-up penalty. Obes Rev. 2019;20:964–82. 30868745 10.1111/obr.12845

[74] Schloemer T, Schröder-Bäck P. Criteria for evaluating transferability of health interventions: a systematic review and thematic synthesis. Implement Sci. 2018;13:88. 29941011 10.1186/s13012-018-0751-8 PMC6019740

[75] Pampel FC, Krueger PM, Denney JT. Socioeconomic disparities in health behaviors. Annu Rev Sociol. 2010;36:349–70. 21909182 10.1146/annurev.soc.012809.102529 PMC3169799

[76] Loring B, Robertson A. Obesity and inequities: guidance for addressing inequities in overweight and obesity. World Health Organization. 2014. https://apps.who.int/iris/bitstream/handle/10665/344619/9789289050487-eng.pdf?sequence=1&isAllowed=y.

[77] Ntais C, Talias MA. Unveiling the value of meta-analysis in disease prevention and control: a comprehensive review. Medicina (Kaunas). 2024;60:1629. 39459416 10.3390/medicina60101629 PMC11509094

[78] Di Cesare M, Sorić M, Bovet P, Miranda JJ, Bhutta Z, Stevens GA, et al. The epidemiological burden of obesity in childhood: a worldwide epidemic requiring urgent action. BMC Med. 2019;17:212. 31760948 10.1186/s12916-019-1449-8 PMC6876113

