# Supplementary material for: Assessing the effectiveness of healthy weight interventions in the early years of childhood: a systematic review and meta-analysis of evidence from high-income countries
Source: Int J Obes (Lond). 2026 Apr 9;50(6):1180–9. doi: 10.1038/s41366-026-02073-8 (PMC13286995; doi:10.1038/s41366-026-02073-8)
Supplement: Supplementary file 1 — Supplement 1 [file 41366_2026_2073_MOESM1_ESM.docx]

**Supplemental Material**

**Assessing the effectiveness of healthy weight interventions in the early years of childhood: A systematic review and meta-analysis of evidence from high-income countries**

Disha Dhar^1^*, Lucy Karwatowska^1^*, Maria Sifaki^1^, Semina Michalopoulou^1^, Claire Stansfield^2^, Jessica Packer^1^**, and Simon J. Russell^1^**

^1^ Great Ormond Street Institute of Child Health, University College London, London, United Kingdom

^2^ UCL Institute of Education, University College London, London, UK

* Joint first authorship

** Joint senior authorship

**Appendix -**

**eAppendix 1.** Supplementary Methods

**Tables -**

**eTable 1.** Preferred Reporting Items for Systematic Reviews and Meta-Analyses (PRISMA) Checklist

**eTable 2.** The Population, Intervention, Comparison, Outcomes and Study design (PICOS) framework used in the current review

**eTable 3.** Search terms and exemplar search strategy

**eTable 4.** Rationale for inclusion/exclusion in meta-analyses

**eTable 5.** Transformations

**eTable 6.** Selected characteristics of included studies

**eTable 7.** A descriptive summary of the participant characteristics and study features of the studies included in the narrative synthesis (k = 40).

**eTable 8.** Meta-analytic associations

**eTable 9.** Sensitivity and subgroup analyses

**eTable 10.** The studies that examined intervention effects by socioeconomic status

**eTable 11.** The certainty of evidence scores based on the Grading of Recommendation, Assessment, Development, and Evaluation (GRADE) framework

**Figures -**

**eFigure 1.** Funnel plot of the effect sizes included in the meta-analysis of BMI z-scores.

**eFigure 2.** Funnel plot of the effect sizes included in the meta-analysis of waist circumference.

**eFigure 3.** Funnel plot of the effect sizes included in the meta-analysis of BMI percentile.

**eFigure 4.** Funnel plot of the effect sizes included in the meta-analysis of percentage body fat.

**eFigure 5.** Funnel plot of the effect sizes included in the meta-analysis of fat-free mass index.

**eFigure 6.** The scores on the National Institute of Health Quality Assessment Tool (Controlled Intervention Studies) for the studies included in the synthesis.

**eFigure 7.** The scores on the National Institute of Health Quality Assessment Tool (Before-After [Pre-Post] Studies) for the studies included in the synthesis.

**Appendix**

**eAppendix 1.** Supplementary Methods

*Outcome measures*

The primary outcomes were weight-related anthropometric measures commonly used to assess adiposity and weight status in children. BMI, calculated from height and weight, is a widely used metric, with standardised zBMI and percentiles allowing for age- and sex-specific comparisons.^2^ Central adiposity is assessed through waist-related measures, including waist circumference and waist-to-hip ratio, both of which are strong indicators of abdominal fat distribution.^2^ Absolute body weight is frequently used in early childhood assessments, while weight z-scores offer standardised comparisons among children of the same age and sex.^2^ Skinfold thickness assesses adiposity by measuring specific subcutaneous fat depots, such as the biceps, triceps, subscapular and abdomen.^3^ Additional measures of adiposity include fat mass index (FMI) and fat-free mass index (FFMI), which differentiate between fat and lean tissue relative to height,^4^ and percentage body fat, which provides the proportion of total body mass composed of fat.^2^ Including diverse outcome measures offers a comprehensive evaluation of both overall and central adiposity in children.

**References**

1. *NICE Evidence Reviews Collection*, in *Evidence review for effectiveness and acceptability of weight management interventions in children and young people living with overweight and obesity: Overweight and obesity management: preventing, assessing and managing overweight and obesity: Evidence review G*. 2025, National Institute for Health and Care Excellence (NICE) Copyright © NICE 2025.: London.

2. Casadei, K. and J. Kiel, *Anthropometric Measurement*, in *StatPearls*. 2025, StatPearls Publishing Copyright © 2025, StatPearls Publishing LLC.: Treasure Island (FL) ineligible companies. Disclosure: John Kiel declares no relevant financial relationships with ineligible companies.

3. Wells, J.C. and M.S. Fewtrell, *Measuring body composition.* Arch Dis Child, 2006. **91**(7): p. 612-7.

4. Shypailo, R.J. and W.W. Wong, *Fat and fat-free mass index references in children and young adults: assessments along racial and ethnic lines.* Am J Clin Nutr, 2020. **112**(3): p. 566-575.

**Tables**

**eTable 1.** *Preferred Reporting Items for Systematic Reviews and Meta-Analyses (PRISMA) Checklist.*

| **Section and Topic** | **Item #** | **Checklist item** | **Location where item is reported** |
| --- | --- | --- | --- |
| **TITLE** | | |  |
| Title | 1 | Identify the report as a systematic review. | 1 pg. |
| **ABSTRACT** | | |  |
| Abstract | 2 | See the PRISMA 2020 for Abstracts checklist. | 2-3 pg. |
| **INTRODUCTION** | | |  |
| Rationale | 3 | Describe the rationale for the review in the context of existing knowledge. | 4-5 pg. |
| Objectives | 4 | Provide an explicit statement of the objective(s) or question(s) the review addresses. | 5 pg. |
| **METHODS** | | |  |
| Eligibility criteria | 5 | Specify the inclusion and exclusion criteria for the review and how studies were grouped for the syntheses. | 8 pg. |
| Information sources | 6 | Specify all databases, registers, websites, organisations, reference lists and other sources searched or consulted to identify studies. Specify the date when each source was last searched or consulted. | 7 pg. |
| Search strategy | 7 | Present the full search strategies for all databases, registers and websites, including any filters and limits used. | eTable 3, Supplement 1 |
| Selection process | 8 | Specify the methods used to decide whether a study met the inclusion criteria of the review, including how many reviewers screened each record and each report retrieved, whether they worked independently, and if applicable, details of automation tools used in the process. | 8-9 pg. |
| Data collection process | 9 | Specify the methods used to collect data from reports, including how many reviewers collected data from each report, whether they worked independently, any processes for obtaining or confirming data from study investigators, and if applicable, details of automation tools used in the process. | 9 pg. |
| Data items | 10a | List and define all outcomes for which data were sought. Specify whether all results that were compatible with each outcome domain in each study were sought (e.g. for all measures, time points, analyses), and if not, the methods used to decide which results to collect. | 9 pg. and eAppendix 1 |
|  | 10b | List and define all other variables for which data were sought (e.g. participant and intervention characteristics, funding sources). Describe any assumptions made about any missing or unclear information. | 10-11 pg. |
| Study risk of bias assessment | 11 | Specify the methods used to assess risk of bias in the included studies, including details of the tool(s) used, how many reviewers assessed each study and whether they worked independently, and if applicable, details of automation tools used in the process. | 10 pg. |
| Effect measures | 12 | Specify for each outcome the effect measure(s) (e.g. risk ratio, mean difference) used in the synthesis or presentation of results. | 10-11 pg. |
| Synthesis methods | 13a | Describe the processes used to decide which studies were eligible for each synthesis (e.g. tabulating the study intervention characteristics and comparing against the planned groups for each synthesis (item #5)). | eTable 2 and eTable 4, Supplement 1 |
|  | 13b | Describe any methods required to prepare the data for presentation or synthesis, such as handling of missing summary statistics, or data conversions. | 10-11 pg. and eTable 5 |
|  | 13c | Describe any methods used to tabulate or visually display results of individual studies and syntheses. | 10-11 pg. |
|  | 13d | Describe any methods used to synthesize results and provide a rationale for the choice(s). If meta-analysis was performed, describe the model(s), method(s) to identify the presence and extent of statistical heterogeneity, and software package(s) used. | 10-11 pg. and eTable 4 |
|  | 13e | Describe any methods used to explore possible causes of heterogeneity among study results (e.g. subgroup analysis, meta-regression). | 10-11 pg. |
|  | 13f | Describe any sensitivity analyses conducted to assess robustness of the synthesized results. | 10-11 pg. |
| Reporting bias assessment | 14 | Describe any methods used to assess risk of bias due to missing results in a synthesis (arising from reporting biases). | 10-11 pg. |
| Certainty assessment | 15 | Describe any methods used to assess certainty (or confidence) in the body of evidence for an outcome. | 10-11 pg. |
| **RESULTS** | | |  |
| Study selection | 16a | Describe the results of the search and selection process, from the number of records identified in the search to the number of studies included in the review, ideally using a flow diagram. | 12 pg. |
|  | 16b | Cite studies that might appear to meet the inclusion criteria, but which were excluded, and explain why they were excluded. | 12 pg., and Figure 1 |
| Study characteristics | 17 | Cite each included study and present its characteristics. | 12-13 pg., and eTable 7 |
| Risk of bias in studies | 18 | Present assessments of risk of bias for each included study. | eFigure 6 and eFigure 7 |
| Results of individual studies | 19 | For all outcomes, present, for each study: (a) summary statistics for each group (where appropriate) and (b) an effect estimate and its precision (e.g. confidence/credible interval), ideally using structured tables or plots. | eTable 6., eTable 8 |
| Results of syntheses | 20a | For each synthesis, briefly summarise the characteristics and risk of bias among contributing studies. | 13-16 pg. |
|  | 20b | Present results of all statistical syntheses conducted. If meta-analysis was done, present for each the summary estimate and its precision (e.g. confidence/credible interval) and measures of statistical heterogeneity. If comparing groups, describe the direction of the effect. | 13-16 pg., eTable 8., Figure 2-6 |
|  | 20c | Present results of all investigations of possible causes of heterogeneity among study results. | 16 |
|  | 20d | Present results of all sensitivity analyses conducted to assess the robustness of the synthesized results. | 17 |
| Reporting biases | 21 | Present assessments of risk of bias due to missing results (arising from reporting biases) for each synthesis assessed. | 17-18 |
| Certainty of evidence | 22 | Present assessments of certainty (or confidence) in the body of evidence for each outcome assessed. | 17-18 |
| **DISCUSSION** | | |  |
| Discussion | 23a | Provide a general interpretation of the results in the context of other evidence. | 19 |
|  | 23b | Discuss any limitations of the evidence included in the review. | 20-21 |
|  | 23c | Discuss any limitations of the review processes used. | 20-21 |
|  | 23d | Discuss implications of the results for practice, policy, and future research. | 19-20 |
| **OTHER INFORMATION** | | |  |
| Registration and protocol | 24a | Provide registration information for the review, including register name and registration number, or state that the review was not registered. | 2 |
|  | 24b | Indicate where the review protocol can be accessed, or state that a protocol was not prepared. | 2 |
|  | 24c | Describe and explain any amendments to information provided at registration or in the protocol. | 2 |
| Support | 25 | Describe sources of financial or non-financial support for the review, and the role of the funders or sponsors in the review. | 2 |
| Competing interests | 26 | Declare any competing interests of review authors. | 2 |
| Availability of data, code and other materials | 27 | Report which of the following are publicly available and where they can be found: template data collection forms; data extracted from included studies; data used for all analyses; analytic code; any other materials used in the review. | 2 |

**eTable 2.** *The Population, Intervention, Comparison, Outcomes and Study design (PICOS) framework used in the current review.*

|  | **Inclusion** | **Exclusion** |
| --- | --- | --- |
| **Participants** | - Preschool children 5 years old and under. | - Includes children 6 years and older. - Children suffering from illnesses or co-morbidities. |
| **Exposure (intervention)** | - Individual and/or community-based programmes or interventions that aim to prevent obesity in early years. - Universal interventions that are multi-component (e.g. diet, physical activity and/or behavioural change) and may be delivered in multiple settings (e.g. in-home or pre-school or childcare or healthcare settings). | - Individual and/or community-based programmes or interventions that do not aim to prevent obesity in early years. - Targeted interventions that are not multi-competent. |
| **Comparison/ study design** | Before-and-after implementation evaluations, real world studies, including:   - Randomised controlled trials (RCTs) or cluster RCTs. - Experiment studies. | Modelling studies, qualitative studies (unless part of an intervention study), and systematic reviews. |
| **Outcome measures** | Any weight-related anthropometric measures (e.g. BMI, BMI-z score, BMI percentile and waist circumference). | No weight-related anthropometric measure. |
| **Geography** | Countries classified as high-income countries by The World Bank. | Countries not classified as high-income by the World Bank. |
| **Languages** | All | NA |
| **Time** | 2011- present | Published prior to 2011 |
| **Publication type** | Intervention studies or randomized controlled trials, with pre- and post-implementation measures. | Conference abstracts, case studies, reviews, editorials, proceedings etc. |

**eTable 3.** *Search terms and exemplar search strategy*

| **SEARCH TERMS** | |
| --- | --- |
| **Database** | **MeSH Terms** |
| PubMed, PsycInfo, Social Policy and Practice, and Health Management Information Consortium | **Nutrition -** Food/ OR Beverages/ OR Food quality/ OR Food preferences/ OR Feeding behaviour/  **Physical activity and other behavioural components -** Exercise/ OR Physical fitness/ OR Sedentary behavior/  **Intervention component -** Weight reduction programs/ OR Health education/ OR Health promotion/ OR Primary prevention/  **Study design -** Pilot projects/ OR Feasibility studies/ OR Program evaluation/ OR Clinical trials/  **Outcome measures -** Obesity/ OR Body mass index/ OR Body weight/ OR Body size/ OR Body composition/ OR Body weight changes/ OR Overweight/ |
| Cochrane Library CENTRAL | Not applicable. |
| Web of Science (SSCI, ESCI), and CINAHL | Not applicable. |
| Scopus, and Trials Register of Promoting Health Interventions | Not applicable. |
| **Free Text Search Terms** | |
| **Concept 1 –**  Participants | 1. child preschool OR preschool* OR pre school* OR kindergarten OR kindergarden OR prekinder* OR pre kinder* OR headstart OR head start OR sure start OR surestart OR family child care home* OR family childcare home* OR infant* OR toddler* OR childcare OR child-care OR child-care OR early learning center* OR early learning centre* OR early child* OR early care and education OR ("child*" AND ("day-care" OR "day-care" OR "daycare")) OR (playgroup* OR play group* OR schools nursery OR (("center" OR "center s" OR "centers" OR "centre" OR "centre s" OR "centres") AND "child, preschool") OR ("child day care centers" OR "child day care centers") |
| **Concept 2 –** Nutrition, physical activity, behavioural intervention | 1. diet* OR dietary OR food* OR snack* OR fruit* OR drink* OR beverage* OR juice* OR vegetable* OR meal* OR nutrition* OR intake* OR consum* OR food quality OR energy density OR eating behavio?r OR healthy eating OR feeding behaviour?r OR food preference* OR health promotion OR eating habit* OR food habit* OR food choice* OR overeat* OR over eat* OR menu planning OR menu choice OR meal choice* OR meal* OR meal planning OR eating habit* 2. physical* activ* OR playtime OR sedentary behavio?r or exercise* OR play* OR physical fitness OR movement OR playtime OR sport* OR play* OR lifestyle behavio?r 3. intervention* OR weight reduction program* OR weight management OR weight control OR program* OR programme* OR project* OR therapeutics OR scheme OR initiative* OR strateg* OR service* OR pilot projects OR feasibility studies OR program evaluation OR health education OR health promotion OR primary prevention OR treat* OR random* OR cluster random* OR trial* OR evaluat* OR effective* OR mixed methods OR impact* |
| **Concept 3 –** Outcome measure | 1. overweight OR obesity OR obes* OR body mass index OR BMI OR bmi z score OR bmi z score OR body mass index z-score OR body mass index z-score OR bmi percentile* OR bmi centile* OR body mass index percentile* OR body mass index centile*  OR adipos*  OR overweight OR healthy weight OR weight loss OR weight gain OR body weight OR body size OR body composition OR body weight changes |
| **Concept 4 –** Study design | 1. pilot project* OR feasibility stud* OR program evaluation OR health education* OR health promotion OR primary prevention or treat* OR random* OR cluster random* OR trial* OR evaluat* OR effective* OR mixed methods OR impact* |

Example of Social Policy and Practice search strategy. For detailed search strategies in other databases, please contact the corresponding author.

| **1** | (((preschool* or pre-school* or pre-school* or kindergar$en or prekinder* or pre-Kinder* or pre Kinder* or headstart or head start or surestart or sure start or family child care home or family childcare home or infant* or toddler* or child care or child-care or childcare or early learning centre or early child* or early care) and education) or ((child* and day care) or daycare or day-care or playgroup* or nurser* or child day care centre*)).mp. [mp=abstract, title, publication type, heading word, accession number] |
| --- | --- |
| **2** | (diet* or dietary or food* or snack* or fruit* or drink* or beverage* or juice* or vegetable* or meal* or nutrition* or intake* or consum* or food quality or food preference* or energy density or lifestyle behavio?r or eating behavio?r or healthy eating or feeding behaviour?r or health promotion or eating habit* or food habit* or food choice* or overeat* or over eat* or weight control or menu planning or menu choice or meal choice* or meal* or meal planning or (food* and consum*) or eating habit*).mp. [mp=abstract, title, publication type, heading word, accession number] |
| **3** | (physical* activ* or playtime or sedentary or exercise* or play* or physical fitness or movement or playtime or sport*).mp. [mp=abstract, title, publication type, heading word, accession number] |
| **4** | (intervention* or weight reduction program* or program* or programme* or project* or therapeutics or scheme or initiative* or strateg* or life style or service* or pilot project* or feasibility stud* or program evaluation or health education* or health promotion or primary prevention or treat* or random* or cluster random* or trial* or evaluat* or effective* or mixed methods or impact*).mp. [mp=abstract, title, publication type, heading word, accession number] |
| **5** | (intervention* or weight reduction programs or program*OR programme* or project* or therapeutics or scheme or initiative* or strateg* or service* or health education or health promotion or primary prevention or health education or health promotion or primary prevention or treat* or therapy* or pilot projects or feasibility studies or program evaluation).mp. [mp=abstract, title, publication type, heading word, accession number] |
| **6** | (random* or cluster random* or trial* or evaluat* or effective* or mixed methods or impact* or Non-Randomized Controlled Trial* or Pragmatic Clinical Trial or controlled Clinical Trial* or randomized controlled trial* or randomized or randomised or clinical trial* or randomly or controlled study or controlled trial* or control group or intervention group or experimental group or comparison group or control school or intervention school or control community or intervention community or intervention condition or control condition or treatment group or control participant or quasi experimental design or experimental condition or treatment condition or pilot projects or feasibility studies or program evaluation or pre test or post test or quasi experiment* or blinded or double blind or fidelity or reach or uptake or implementation or intervention effect* or program effect* or programme effect* or proof of concept* or outcome study).mp. [mp=abstract, title, publication type, heading word, accession number] |
| **7** | 5 and 6 |
| **8** | 2 or 3 |
| **9** | 4 or 7 |
| **10** | 8 and 9 |
| **11** | (obesity or obes* or body mass index or BMI or bmi z score or bmi z score or body mass index z-score or body mass index z-score or bmi percentile* or bmi centile* or body mass index percentile* or body mass index centile* or body weight or body size or weight* or waist or adipos* or body composition or overweight or body weight changes or healthy weight or weight loss or weight gain).mp. [mp=abstract, title, publication type, heading word, accession number] |
| **12** | 1 and 10 and 11 |
| **13** | limit 12 to yr="2021 -Current" |

**eTable 4.** *Rationale for inclusion/exclusion in meta-analyses.*

| **Author (Year)** | **Relevant outcomes** | **Rationale for inclusion/exclusion** |
| --- | --- | --- |
| **Included in meta-analyses** | | |
| *Alexandrou et al. (2023)* | zBMI | *Study design*: RCT  *Data provided*: Means, SDs and Ns for intervention and control groups at baseline and follow-up.  *Transformations*: Means, SDs and Ns used to calculate standardised mean change. Standardised mean change in intervention and control groups used to calculate standardised mean difference. |
| *Alkon et al. (2014)* | zBMI | *Study design*: Cluster RCT  *Data provided*: Means, SDs and Ns for intervention and control groups at baseline and follow-up.  *Transformations*: Means, SDs and Ns used to calculate standardised mean change. Standardised mean change in intervention and control groups used to calculate standardised mean difference. |
| *Campbell et al. (2013)* | zBMI | *Study design*: Cluster RCT  *Data provided*: Means, SDs and Ns for intervention and control groups at baseline and follow-up.  *Transformations*: Means, SDs and Ns used to calculate standardised mean change. Standardised mean change in intervention and control groups used to calculate standardised mean difference. |
| *Davis et al. (2016)* | zBMI | *Study design*: Cluster RCT  *Data provided*: Means, 95% CIs and Ns for intervention and control group at baseline and follow-up.  *Transformations*: 95% CIs transformed to SD. Means, SDs and Ns used to calculate standardised mean change. Standardised mean change in intervention and control groups used to calculate standardised mean difference. |
| *De Coen et al. (2012)* | zBMI | *Study design*: Cluster RCT  *Data provided*: Means, SDs and Ns for intervention and control groups at baseline and follow-up.  *Transformations*: Means, SDs and Ns used to calculate standardised mean change. Standardised mean change in intervention and control groups used to calculate standardised mean difference. |
| *Döring et al. (2016)* | Waist circumference | *Study design*: Cluster RCT  *Data provided*: Means, SDs and Ns for intervention and control groups at baseline and follow-up.  *Transformations*: Means, SDs and Ns used to calculate standardised mean change. Standardised mean change in intervention and control groups used to calculate standardised mean difference. |
| *French et al. (2018)* | zBMI  BMI percentiles  Waist circumference | *Study design*: RCT  *Data provided*: Means, SDs and Ns for intervention and control groups at baseline and follow-up.  *Transformations*: Means, SDs and Ns used to calculate standardised mean change. Standardised mean change in intervention and control groups used to calculate standardised mean difference. |
| *French et al. (2023)* | zBMI  BMI percentiles | *Study design*: RCT  *Data provided*: Means, SDs and Ns for intervention and control groups at baseline and follow-up.  *Transformations*: Means, SDs and Ns used to calculate standardised mean change. Standardised mean change in intervention and control groups used to calculate standardised mean difference. |
| *Haines et al. (2013)* | zBMI | *Study design*: RCT  *Data provided*: Means, SDs and Ns for intervention and control groups at baseline and follow-up.  *Transformations*: Means, SDs and Ns used to calculate standardised mean change. Standardised mean change in intervention and control groups used to calculate standardised mean difference. |
| *Haines et al. (2018)* | Fat mass index | *Study design*: RCT  *Data provided*: Means, SDs and Ns for intervention and control groups at baseline and follow-up.  *Transformations*: Means, SDs and Ns used to calculate standardised mean change. Standardised mean change in intervention and control groups used to calculate standardised mean difference. |
| *Hodgkinson et al. (2019)* | zBMI | *Study design*: Cluster RCT  *Data provided*: Means, SEs and Ns for intervention and control group at baseline and follow-up.  *Transformations*: SE transformed to SD. Means, SDs and Ns used to calculate standardised mean change. Standardised mean change in intervention and control groups used to calculate standardised mean difference. |
| *Iaia et al. (2017)* | zBMI | *Study design*: Cluster RCT  *Data provided*: Means, 95% CIs and Ns for intervention and control group at baseline and follow-up.  *Transformations*: 95% CIs transformed to SD. Means, SDs and Ns used to calculate standardised mean change. Standardised mean change in intervention and control groups used to calculate standardised mean difference. |
| *Karssen et al. (2022)* | zBMI | *Study design*: RCT  *Data provided*: Means, SEs and Ns for intervention and control group at baseline and follow-up.  *Transformations*: SE transformed to SD. Means, SDs and Ns used to calculate standardised mean change. Standardised mean change in intervention and control groups used to calculate standardised mean difference. |
| *Lanigan et al. (2013)* | zBMI  Waist circumference | *Study design*: RCT  *Data provided*: Means, SDs and Ns for intervention and control groups at baseline and follow-up.  *Transformations*: Means, SDs and Ns used to calculate standardised mean change. Standardised mean change in intervention and control groups used to calculate standardised mean difference. |
| *Morshed et al. (2019)* | zBMI  BMI percentiles | *Study design*: RCT  *Data provided*: Means, SEs and Ns for intervention and control group at baseline and follow-up.  *Transformations*: SE transformed to SD. Means, SDs and Ns used to calculate standardised mean change. Standardised mean change in intervention and control groups used to calculate standardised mean difference. |
| *Natale et al. (2014)* | zBMI | *Study design*: RCT  *Data provided*: Means, 95% CIs and Ns for intervention and control group at baseline and follow-up.  *Transformations*: 95% CIs transformed to SD. Means, SDs and Ns used to calculate standardised mean change. Standardised mean change in intervention and control groups used to calculate standardised mean difference. |
| *Natale et al. (2017)* | BMI percentiles | *Study design*: RCT  *Data provided*: Standardised betas and 95% CIs.  *Transformations*: 95% CIs transformed to SD. Standardised beta transformed into Cohen’s d (i.e. standardised mean difference) using SD and Ns. |
| *Nemet et al. (2011a)* | BMI percentiles | *Study design*: Cluster RCT  *Data provided*: Mean change, SEs and Ns for intervention and control groups.  *Transformations*: SEs transformed to SD. Mean changes were standardised using SD to calculate standardised mean change. Standardised mean change in intervention and control groups used to calculate standardised mean difference. |
| *Nemet et al. (2011b)* | BMI percentiles | *Study design*: Cluster RCT  *Data provided*: Mean change, SEs and Ns for intervention and control groups.  *Transformations*: SEs transformed to SD. Mean changes were standardised using SD to calculate standardised mean change. Standardised mean change in intervention and control groups used to calculate standardised mean difference. |
| *Nyström et al. (2017)* | Fat mass index  Fat-free mass index | *Study design*: RCT  *Data provided*: Mean change, SEs and Ns for intervention and control groups.  *Transformations*: SEs transformed to SD. Mean changes were standardised using SD to calculate standardised mean change. Standardised mean change in intervention and control groups used to calculate standardised mean difference. |
| *Nyström et al. (2018)* | Fat mass index  Fat-free mass index | *Study design*: RCT  *Data provided*: Mean change, SEs and Ns for intervention and control groups.  *Transformations*: SEs transformed to SD. Mean changes were standardised using SD to calculate standardised mean change. Standardised mean change in intervention and control groups used to calculate standardised mean difference. |
| *Olsen et al. (2020)* | zBMI  Fat mass index  Fat-free mass index  Waist circumference | *Study design*: RCT  *Data provided*: Means, SDs and Ns for intervention and control groups at baseline and follow-up.  *Transformations*: Means, SDs and Ns used to calculate standardised mean change. Standardised mean change in intervention and control groups used to calculate standardised mean difference. |
| *Østbye et al. (2012)* | zBMI | *Study design*: RCT  *Data provided*: Mean change, SDs and Ns for intervention and control groups.  *Transformations*: Mean changes were standardised using SD to calculate standardised mean change. Standardised mean change in intervention and control groups used to calculate standardised mean difference. |
| *Peñalvo et al. (2015)* | zBMI  Waist circumference | *Study design*: Cluster RCT  *Data provided*: Means, SDs and Ns for intervention and control groups at baseline and follow-up.  *Transformations*: Means, SDs and Ns used to calculate standardised mean change. Standardised mean change in intervention and control groups used to calculate standardised mean difference. |
| *Puder et al. (2011)* | Waist circumference  Fat mass index | *Study design*: Cluster RCT  *Data provided*: Means, SDs and Ns for intervention and control groups at baseline and follow-up.  *Transformations*: Means, SDs and Ns used to calculate standardised mean change. Standardised mean change in intervention and control groups used to calculate standardised mean difference. |
| *Sanders et al. (2021)* | zBMI | *Study design*: Cluster RCT  *Data provided*: Standardised betas and 95% CIs.  *Transformations*: 95% CIs transformed to SD. Standardised beta transformed into Cohen’s d (i.e. standardised mean difference) using SD and Ns. |
| *Vaughn et al. (2020)* | zBMI | *Study design*: Cluster RCT  *Data provided*: Means, SDs and Ns for intervention and control groups at baseline and follow-up.  *Transformations*: Means, SDs and Ns used to calculate standardised mean change. Standardised mean change in intervention and control groups used to calculate standardised mean difference. |
| *Verbestel et al. (2014)* | zBMI | *Study design*: Cluster RCT  *Data provided*: Means, SDs and Ns for intervention and control groups at baseline and follow-up.  *Transformations*: Means, SDs and Ns used to calculate standardised mean change. Standardised mean change in intervention and control groups used to calculate standardised mean difference. |
| *Ward et al. (2020)* | BMI percentiles | *Study design*: Cluster RCT  *Data provided*: Means, SDs and Ns for intervention and control groups at baseline and follow-up.  *Transformations*: Means, SDs and Ns used to calculate standardised mean change. Standardised mean change in intervention and control groups used to calculate standardised mean difference. |
| *Zask et al. (2012)* | zBMI  Waist circumference | *Study design*: Cluster RCT  *Data provided*: Means, SEs and Ns for intervention and control group at baseline and follow-up.  *Transformations*: SE transformed to SD. Means, SDs and Ns used to calculate standardised mean change. Standardised mean change in intervention and control groups used to calculate standardised mean difference. |
| **Excluded from meta-analyses** | | |
| *Cloutier et al. (2015)* | BMI percentile  Prevalence of overweight  Prevalence of obesity | *Study design*: experimental  *Reason for exclusion*: no control group. |
| *Enö Persson et al. (2018)* | BMI  Prevalence of overweight  Prevalence of obesity | *Study design*: RCT  *Reason for exclusion*: did not run meta-analyses on weight status categories or BMI. |
| *Lumeng et al. (2017)* | zBMI  Prevalence of obesity AND Overweight or obese | *Study design*: cluster RCT  *Reason for exclusion*: no waitlist/no intervention control group. |
| *Sharma et al. (2019)* | zBMI  BMI percentiles  Prevalence of overweight and obesity | *Study design*: experimental  *Reason for exclusion*: no control group. |
| *Steenbock et al. (2019)* | Percentage body fat  Prevalence of overweight and obesity | *Study design*: experimental  *Reason for exclusion*: no control group. |
| *Stookey et al. (2017)* | zBMI  BMI percentiles  Weight categories | *Study design*: cluster RCT  *Reason for exclusion*: no waitlist/no intervention control group. |
| *Strauß et al. (2011)* | Prevalence of overweight and obesity | *Study design*: Cluster RCT  *Reason for exclusion*: did not run meta-analyses on weight status categories. |
| *Woo et al. (2017)* | zBMI | *Study design*: experimental  *Reason for exclusion*: no control group. |
| *Yin et al. (2022)* | zBMI  BMI percentiles  Body weight z-score | *Study design*: cluster RCT  *Reason for exclusion*: no waitlist/no intervention control group. |
| *van de Kolk et al. (2019)* | zBMI | *Study design*: experimental  *Reason for exclusion*: no control group. |

*Note. Abbreviations: CI = confidence intervals; N = number of individuals; SD = standard deviation; SE = standard error.*

**eTable 5.** *Transformations*

| **Raw effect size type** | **Formulae** |
| --- | --- |
| 95% confidence intervals | $SD=\sqrt{N}\times\frac{uCI-lCI}{3.92}$ |
| Standard error | $SD=SE\times\sqrt{N}$ |
| Mean change | $SMC= \frac{MC}{SD}$ |
| Standardised beta | $d=\beta\times{SD}^{EXP}$ |

*Note. Abbreviations:* β = standardised beta; uCI = upper 95% confidence interval; lCI = lower 95% confidence interval; MC = mean change; N = sample size; SE = standard error; SD = standard deviation; SD^EXP^ = standard deviation of the exposure; SMC = standardised mean change.

**eTable 6.** *Selected characteristics of included studies*

| **Author (Year)** | **Country** | **Study design** | **Sample details**  **(sample size and ages)** | **Intervention duration (months)** | **Intervention description** | **Follow-up (months)** | **Outcomes measured** |
| --- | --- | --- | --- | --- | --- | --- | --- |
| *Alexandrou et al. (2023)* | Sweden | RCT | 552  Children  (2.5 - 3 years) | 6 | Intervention delivered via a smartphone application. New information about healthy eating and physical activity introduced biweekly, with parents also receiving weekly personalized feedback. Parents could contact a dietitian or a psychologist to ask questions. | 6 | zBMI |
| *Alkon et al. (2014)* | USA | Cluster RCT | 552  Children  (3 - 5 years) | 7 | Intervention delivered in child-care health centres and included five one-hour workshops for staff, parent sessions on 'Raising Healthy Kids,' monthly on-site consultations, and additional support via phone or email. Posters and informational sheets on nutrition and physical activity were also distributed. | 14 | zBMI; Prevalence of overweight; Prevalence of obesity |
| *Campbell et al. (2013)* | Australia | Cluster RCT | 542  Infants  (4 months) | 15 | Intervention delivered by experienced dietitian, comprising of six sessions at three-month intervals during the regular meeting time of the first-time parents' group. A newsletter reinforcing key messages was sent to participants between session. | 5, 16 | zBMI |
| *Cloutier et al. (2015)* | USA | Experimental | 467  Infants  (4 months) | 12 | Intervention delivered by primary care clinicians and nurses and included regularly scheduled clinic visits. Staff provided educational materials and toolkits to mothers that target four specific obesogenic behaviours. | 12 | BMI percentiles |
| *Davis et al. (2016)* | USA | Cluster RCT | 980  Children  (< 4 years) | 18 | Intervention delivered at Head Start centres by trained staff and professionals. It included a curriculum promoting and encouraging healthy behaviours and serving practices within the centres. Families received take-home materials and participated in events; local grocery stores supported the initiative and healthcare providers promoted healthy habits during routine visits and participated in family events. | 7, 12, 19 | zBMI |
| *De Coen et al. (2012)* | Belgium | Cluster RCT | 1,589  Children  (3 - 6 years) | 24 | Intervention delivered in various settings including schools, home, community sites. Intervention included, "Healthy Weeks" and curriculums in school delivered by trained teachers, school policy changes (e.g., water fountains, fruit availability), parent education with health tips, five informational letters, access to a website with recipes and advice, and tailored feedback. | 19 | zBMI |
| *Döring et al. (2016)* | Sweden | Cluster RCT | 1,148  Infants  (9 - 10 months) | 39 | Intervention delivered in child health centres by trained nurses. Directed towards first-time parents attending regular check-ups. Families participated in 1 group session and 8 individual sessions. Intervention nurses assisted parents in promoting healthy habits in their children and in changing their own health behaviours, if needed. | 39 | BMI; Waist circumference; Prevalence of overweight |
| *Enö Persson et al. (2018)* | Sweden | RCT | 1,091  Infants  (9 - 10 months) | 39 | Intervention delivered in child health centres by trained nurses. Directed towards first-time parents attending regular check-ups. Families participated in 1 group session and 8 individual sessions. Intervention nurses assisted parents in promoting healthy habits in their children and in changing their own health behaviours, if needed. | 12 | BMI; Prevalence of overweight; Prevalence of obesity |
| *French et al. (2018)* | USA | RCT | 534  Children  (2 - 4 years) | 36 | Intervention delivered via telephone as well as by home visiting, and community-based parenting classes. Monthly 1-hour home visits and intervals with telephone check-in calls between home visits and weekly parenting classes for 12 weeks in the communities. Referrals to community resources for healthy foods and physical activity opportunities were embedded in the home visiting and parenting class components. | 12, 24, 36 | zBMI; BMI; BMI percentile; Waist circumference; Body weight; Triceps skinfolds |
| *French et al. (2023)* | USA | RCT | 338  Children  (2 - 4 years) | 36 | Intervention delivered via telephone as well as by home visiting, and community-based parenting classes. Monthly 1-hour home visits and intervals with telephone check-in calls between home visits and weekly parenting classes for 12 weeks in the communities. Referrals to community resources for healthy foods and physical activity opportunities were embedded in the home visiting and parenting class components. | 66 | zBMI; BMI; BMI percentile; Prevalence of obesity |
| *Haines et al. (2013)* | USA | RCT | 111  Children  (2 - 5 years) | 6 | Intervention delivered by four in-home coaching visits, four follow-up phone calls, mailed educational materials and incentives, and weekly text messages. | 6 | zBMI; BMI |
| *Haines et al. (2018)* | Canada | RCT | 42  Children  (1.5 - 5 years) | 6 | Intervention delivered via 1-hour home visits by four health educators focusing on behavioral goals. Families assessed their current habits and set behaviours change goals and were sent weekly emails that were tailored to the behaviour change goal set by the family. These emails included strategies to support behaviour change. | 6 | Percentage body fat |
| *Hodgkinson et al. (2019)* | UK | Cluster RCT | 80  Children  (2 years +) | 6 | Intervention delivered in Sure Start Early Years’ Centres by trained staff to educate families about eating healthy and being active. A home-based Healthy Heroes program, to promote healthy habits via interactive activities led by trained Centre staff. Delivered over six months in group and one-on-one sessions. | 24 | zBMI |
| *Iaia et al. (2017)* | Italy | Cluster RCT | 361  Children  (3 years +) | 6 | Intervention delivered by two 20-mins face-to-face motivational interviews with parents and provided information tools for parents and teachers displayed in waiting rooms of paediatric clinics and in childcare centre halls. Teachers got a 10-h-long training to promote healthy behaviours into their annual educational timetables. Children and teachers also engaged in learning experiences (~1 h per day). We offered to control children's parents routine healthcare advice during child health visits. | 12, 24 | zBMI; BMI |
| *Karssen et al. (2022)* | The Nether-lands | RCT | 333  Infants  (5 - 15 months) | 12 | Intervention delivered via stand-alone, easy-to-use app consisting of modules about healthy parenting practices. Information in the lessons was presented in an engaging and easy-to-comprehend way, with challenges consisted of exercises that prompted parents to apply the information from the lessons in their day-to-day life. | 6, 12 | zBMI |
| *Lanigan et al. (2013)* | UK | RCT | 85  Children  (1 - 5 years) | 6 | Intervention delivered via by weekly two-hour sessions, consisting of art workshops, music and movement, healthy snack time, and themed educational activities. The first 12 weeks focused on teaching parents about healthy lifestyle principles, while engaging families in creative projects and culturally relevant physical activities. The second half emphasised practical skills such as cooking, portion control, label reading, and active play, to reinforce and apply earlier lessons. Families were provided with materials to support continued learning at home and collaboratively created a recipe book. | 6, 12 | zBMI; BMI; Waist circumference; Body weight; Sum of skinfolds |
| *Lumeng et al. (2017)* | USA | Cluster RCT | 697  Not reported | 7 (repeated over 4 academic years (Sep to May) | Intervention delivered messages to preschoolers and their parents through a classroom component featuring six lessons. The parent component included eight 75-minute sessions incorporating cooking activities and focused on building knowledge, self-efficacy, and practical skills. Additional, for IYS group, 60 preschool classroom lessons followed by small group activities on self-regulation, problem-solving, and prosocial behaviour. The parent component was delivered via video vignettes in 14 group sessions or 10 home visits, supported by homework and follow-up phone calls to reinforce learning. | 7, 19, 31 | zBMI; Prevalence of overweight and obesity; Prevalence of obesity |
| *Morshed et al. (2019)* | USA | RCT | 230  Children  (2-4 years) | 24 | Intervention delivered by a lifestyle program embedded within the standard Parents as Teachers (PAT) curriculum, fostered maternal–child interaction and encouraged mothers to model healthy eating and physical activity behaviours. Guided by Social Cognitive Theory, the intervention addressed behavioral change through inter and intra-personal, and home environment factors. | 12, 24 | zBMI; BMI percentiles; Prevalence of overweight |
| *Natale et al. (2014)* | USA | RCT | 307  Children  (2 - 5 years) | 6 | Intervention delivered by trained teachers and parents were provided with a monthly educational dinner in which nutrition and physical activity were discussed, monthly newsletters, and at-home activities. | 3, 6, 12 | zBMI; Weight *z-*score |
| *Natale et al. (2017)* | USA | RCT | 1,211  Children  (2 - 5 years) | 24 | Intervention delivered in childcare centres that implemented several health-focused policies, including promoting water as the primary beverage, providing only low-fat cow’s milk (<1% fat), limiting juice to once a week, serving fresh fruits or vegetables daily during meals and snacks, encouraging over 60 minutes of physical activity per day, and restricting screen time to less than 30 minutes per week. In addition, parents and teachers received education on a range of nutrition-related topics. | 9, 12, 24 | BMI percentiles |
| *Nemet et al. (2011a)* | Israel | Cluster RCT | 342  Children  (4.2 - 6.5 years) | 12 | Intervention delivered in preschools, with trained teachers delivering content on nutrition, physical activity, and cooking methods, and information on fast-food versus home cooking. Moreover, all intervention children participated in 45 minutes per day of exercise training and parents invited for “Healthy Day Festival”, discussing key topics. | 12 | BMI; BMI percentiles; body weight |
| *Nemet et al. (2011b)* | Israel | Cluster RCT | 725  Children  (3.8 - 6.8 years) | 12 | Intervention delivered in preschools, with trained teachers delivering content on nutrition, physical activity, and cooking methods, and information on fast-food versus home cooking. Moreover, all intervention children participated in 45 minutes per day of exercise training and parents invited for “Healthy Day Festival”, discussing key topics. | 12 | BMI; BMI percentiles; body weight |
| *Nyström et al. (2017)* | Sweden | RCT | 315  Children  (4.5 years) | 6 | Intervention delivered via a smartphone application. New information about healthy eating and physical activity introduced biweekly, with parents also receiving weekly personalized feedback. Parents could contact a dietitian or a psychologist to ask questions. | 6 | Fat mass index; Fat-free mass index; body weight |
| *Nyström et al. (2018)* | Sweden | RCT | 315  Children  (4.5 years) | 6 | Intervention delivered via a smartphone application. New information about healthy eating and physical activity introduced biweekly, with parents also receiving weekly personalized feedback. Parents could contact a dietitian or a psychologist to ask questions. | 12 | Fat mass index; Fat-free mass index; body weight |
| *Olsen et al. (2020)* | Denmark | RCT | 963  Children  (2 - 6 years) | 15 | Intervention delivered by motivational interviewing which tailored to the needs of the family. Sessions focused on how to optimize diet and physical activity habits, how to reduce stress, improve sleep quality and quantity of the child. They took place approximately every 4 months. Families were also invited to bi-weekly cooking classes and play sessions. | 15 | zBMI; Waist circumference; Waist-hip-ratio; Fat mass index; Fat-free mass index; Sum of four skinfolds |
| *Østbye et al. (2012)* | USA | RCT | 400  Children  (2 - 5 years) | 8 | Intervention delivered via monthly 20–30-minute telephone coaching session using motivational interviewing and 8 monthly kits, which included child activities and incentives reinforcing the month's topic. Coaching aimed to improve parenting skills, techniques for stress management, and education about healthy behaviours. | 12 | zBMI |
| *Peñalvo et al. (2015)* | Spain | Cluster RCT | 2,062  Children  (3 - 5 years) | 36 | Intervention delivered in preschool and participants were exposed to 3, 2, or 1 year, depending on their starting grade. Over the academic year, teachers delivered the intervention through for a minimum of 20 h for the diet, physical activity, and human body components and a minimum of 10 h for the emotion management component. Also, the intervention included activities for the family over the weekends, and strategies involving the whole school environment, such as an annual health fair. | 12, 24, 36 | zBMI; BMI; Waist circumference; Prevalence of overweight; Prevalence of obesity; Subscapular skinfold z-score; Triceps skinfold z-score |
| *Puder et al. (2011)* | Switzerland | Cluster RCT | 652  Not reported | 10 | Intervention delivered in preschools that included four 45-minute sessions per week aimed at improving aerobic fitness and coordination, 22 lessons covering healthy nutrition, media use, and sleep. Nutritional messages, based on the Swiss Society of Nutrition's five recommendations, were reinforced at home, children received a new, playful physical activity or nutrition card every other week. Preschools also promoted healthy snacks during recess, healthy birthday treats, and offered only water and nutritious food in the classroom. | 10 | BMI; Waist circumference; Percentage body fat; Prevalence of overweight; Sum of four skinfolds |
| *Sanders et al. (2021)* | USA | Cluster RCT | 865  Infants  (2 months +) | 24 | Interventions delivered via visits to the health-care centre that took place at regular time points. Intervention included parent educational toolkit, including developmentally tailored booklets at each well-child visit. Booklets were designed with low literacy principles to target age specific behavioural goals, with a focus on child nutrition and physical activity; and provider training in health communication. | 4, 6, 9, 12, 15, 18, 24 | zBMI; Prevalence of overweight |
| *Sharma et al. (2019)* | USA | Experimental | 670  Children  (3 - 5 years) | 24 | Intervention delivered through 3 components; “It’s Fun to be Healthy!” a nutrition and gardening-based curriculum, developmentally appropriate structured, indoor, and outdoor physical activities, and parent tip-sheets including recipes, meal plans, parent-child activities, and recommendations for preschoolers’ diet, physical activity, and screen time. | 24 | zBMI; BMI percentiles; Prevalence of overweight and obesity |
| *Steenbock et al. (2019)* | Germany | Experimental | 831  Children  (3 - 6 years) | 12 | The intervention is comprised of 5 modules, 3 focusing on children, 1 on parental participation, and 1 on promoting health among staff. Modules concerned physical activity, nutrition, and sedentary time. | 12 | Percentage body fat; Prevalence of overweight and obesity |
| *Stookey et al. (2017)* | USA | Cluster RCT | 902  Children  (2 - 5 years) | 24 | Intervention delivered in childcare centres. Each centre received specific feedback on how to address its needs. A nutrition workshop addressed ideas for seasonal menu planning, child nutrition education resources for parents, and policies for food for holidays or celebrations. A physical activity workshop addressed how to integrate age-appropriate physical activity and academic learning for preschoolers. These were additional to the standard care, which was received by both the intervention and control clusters. | 12, 24 | zBMI; BMI percentiles; Prevalence of overweight and obesity |
| *Strauß et al. (2011)* | Germany | Cluster RCT | 2,658  Children  Not reported | 24 | Intervention delivered in kindergarten, and staff provided education about healthy nutrition, and narrated stories to children through which they learnt about the food groups and the foundation of a healthy diet. Preschool teachers were given a handbook, a CD with Tiger-Songs, Poster, materials for the parents. | 9, 20 | Prevalence of overweight; Prevalence of obesity |
| *van de Kolk et al. (2019)* | The Nether-lands | Experimental | 191  Children  (2 - 4 years) | 14 | Intervention delivered in various settings including preschool, home, and community sites. At preschool, inspirational session with a physical activity (PA) expert, three interactive training sessions on PA, nutrition, and positive child-rearing, followed by coaching sessions, and distribution of activity cards to help teachers integrate PA and nutrition into the curriculum. Preschools also received play materials, nutrition resources, and complementary fruit and vegetable deliveries. The family component supported families in adopting healthy eating and PA habits, while the community component aimed to strengthen connections between organizations involved in promoting young children’s nutrition and physical activity. | 8, 15 | zBMI |
| *Vaughn et al. (2020)* | USA | Cluster RCT | 853  Children  (3 - 4 years) | 8 | Intervention delivered through classroom activities and targeted early care and education (ECE) providers and parents as key influencers. Featuring educational posters, classroom activities, and parent engagement tools. Delivered in four 6-week units, including classroom posters, classroom activities, and at-home parent prompts and materials such as Family Guide magazines and activity trackers. In wave 2, new tools were added to boost parent engagement. The program concluded with a celebration event for children and families. To support implementation, ECE staff received two trainings and three check-ins by interventionists for guidance, technical assistance, and material distribution. | 9 | zBMI; BMI |
| *Verbestel et al. (2014)* | Belgium | Cluster RCT | 203  Children  (9 - 24 months) | 12 | Intervention delivered in day-care child centres to promote health behaviours. Strategies included persuasive communication, tailoring, and consciousness-raising. The program consisted of two components: a colourful A3 poster with stickers representing five target behaviours, distributed bi-monthly with accompanying letters, and personalized feedback forms based on parents' baseline reports of their children's dietary and activity behaviours. | 12 | zBMI |
| *Ward et al. (2020)* | USA | Cluster RCT | 496  Children  (1.5 - 4 years) | 9 | Intervention delivered in Family Child Care Homes (FCCH), and used strategies like guided practice, self-evaluation, and motivational interviewing to drive behaviours change. The intervention included three modules, focused on provider health, the FCCH environment, and business practices, delivered through workshops, home visits, and follow-up contacts by trained health coaches. Providers were also encouraged to share educational materials with families to support consistency between care and home environments. | 9 | BMI; BMI percentiles |
| *Woo et al. (2017)* | USA | Experimental | 1,461  Children  (2 - 5 years) | 24 | Intervention delivered in clinics by trained staff, and they provided new educational materials and consistent health messaging to parents. Parent counselling sessions addressed various healthy behaviours and staff participated in cross-sector collaborations to strengthen referrals and community support for healthy behaviours. | 24 | zBMI |
| *Yin et al. (2022)* | USA | Cluster RCT | 325  Children  (3 years) | 8 | Intervention delivered in low-income, minority preschools as well as home. All staff received 11–15 hours of training prior to implementation and the intervention boosted parent participation by delivering bilingual, peer-led education sessions, using culturally relevant visuals and demonstrations. Peer educators—bilingual parents with volunteer experience—were trained and compensated to lead eight monthly 15-20 minute sessions. Parents also received newsletters, family health challenges, take-home activity bags, and three home visits by Head Start staff to support goal setting and home environment changes. | 8 | zBMI; BMI; BMI percentiles; *z*-Weight score |
| *Zask et al. (2012)* | Australia | Cluster RCT | 498  Children  (3 - 6 years) | 10 | Intervention delivered in preschools and included, twice-weekly sessions regarding physical activity and healthy eating for children and modification to playgrounds to support active free play. Staff used stories, role-play, taste testing, and costumes to teach children about healthy eating, reinforced with simple, consistent messages and staff role modelling. Parents were engaged through workshops, as well as monthly newsletters with healthy eating and activity tips, and received a food education DVD. | 10 | zBMI; Waist circumference; Prevalence of overweight |
| *Note.* Waist circumference, sum of skinfolds and triceps skinfolds measured in centimetres; sum of four skinfolds measured in millimetres; weight measured in kilograms. *Abbreviations.* BMI = body mass index; zBMI = body mass index z-score; UK = United Kingdom; USA = United States of America; RCT = randomised control trial. | | | | | | | |

**eTable 7.** *A descriptive summary of the participant characteristics and study features of the studies included in the narrative synthesis (k = 40).*

| **Characteristic** | **Overall** **k = 40** |
| --- | --- |
| **Final sample** | 674 (42 – 2,658) |
| **Country** |  |
| United States of America | 18 (45%) |
| Sweden | 5 (13%) |
| Australia | 2 (5%) |
| Belgium | 2 (5%) |
| Germany | 2 (5%) |
| Israel | 2 (5%) |
| Netherlands | 2 (5%) |
| United Kingdom | 2 (5%) |
| Canada | 1 (3%) |
| Denmark | 1 (3%) |
| Italy | 1 (3%) |
| Spain | 1 (3%) |
| Switzerland | 1 (3%) |
| **Year** |  |
| 2011 | 4 (10%) |
| 2012 | 3 (8%) |
| 2013 | 3 (8%) |
| 2014 | 3 (8%) |
| 2015 | 2 (8%) |
| 2016 | 2 (5%) |
| 2017 | 6 (15%) |
| 2018 | 4 (10%) |
| 2019 | 5 (13%) |
| 2020 | 3 (8%) |
| 2021 | 1 (3%) |
| 2022 | 2 (5%) |
| 2023 | 2 (5%) |
| **Study design** |  |
| Cluster randomised control trial | 20 (50%) |
| Randomised control trial | 15 (38%) |
| Experimental | 5 (12%) |
| **Interventional format*** |  |
| In-person | 29 (78%) |
| Mixed | 4 (11%) |
| Digital | 3 (8%) |
| Telephone | 1 (3%) |
| **Outcomes**** |  |
| zBMI | 27 (68%) |
| Weight status categories | 14 (35%) |
| BMI | 14 (35%) |
| BMI percentiles | 11 (28%) |
| Body weight measures | 8 (20%) |
| Waist measures | 8 (20%) |
| Skinfolds measures | 5 (13%) |
| Percentage body fat | 3 (8%) |
| Fat-free mass index | 3 (8%) |
| Fat mass index | 2 (5%) |

*Note.* *Studies included multiple intervention formats; **Studies reported multiple outcomes so total is greater than number of studies included.

**eTable 8.** *Meta-analytic associations*

| **Outcome** | ***k*** | ***ES*** | **N** | **SMD** | **SE** | **lbCI** | **ubCI** | ***I^2^*** |
| --- | --- | --- | --- | --- | --- | --- | --- | --- |
| zBMI | 21 | 39 | 10,849 | -0.085 | 0.027 | -0.140 | -0.029 | 59.32 |
| Waist circumference | 7 | 11 | 3,902 | -0.186 | 0.089 | -0.361 | -0.011 | 77.26 |
| BMI percentiles | 7 | 11 | 3,975 | 0.066 | 0.052 | -0.045 | 0.178 | 75.60 |
| Percentage body fat | 3 | 4 | 1,095 | -0.159 | 0.067 | -0.290 | -0.028 | <0.001 |
| Fat-free mass index | 3 | 3 | 919 | 0.170 | 0.081 | 0.012 | 0.328 | <0.001 |

*Note.* k = number of studies; ES = effect size; N = sample size; SMD = standardized mean difference; SE = standard error; lbCI =lower bound confidence interval; ubCI = upper bound confidence interval; I^2^ = heterogeneity

**eTable 9.** *Moderator analyses*

| **Measurement category** | **Estimate** | **SE** | **lbCI** | **ubCI** |
| --- | --- | --- | --- | --- |
| **zBMI**  *Intercept* | -0.032 | 0.053 | -0.137 | 0.072 |
| *Intervention length* | -0.003 | 0.003 | -0.008 | 0.002 |
| **Waist circumference**  *Intercept* | -0.324 | 0.140 | -0.598 | -0.050 |
| *Intervention length* | 0.006 | 0.005 | -0.003 | 0.015 |
| **BMI percentiles**  *Intercept* | 0.028 | 0.164 | -0.294 | 0.350 |
| *Intervention length* | 0.001 | 0.006 | -0.011 | 0.014 |
| **Percentage body fat**  *Intercept* | -0.306 | 0.305 | -0.904 | 0.292 |
| *Intervention length* | 0.013 | 0.027 | -0.040 | 0.066 |
| **Fat-free mass index**  *Intercept* | 0.222 | 0.188 | -0.146 | 0.591 |
| *Intervention length* | -0.006 | 0.021 | -0.046 | 0.033 |

*Note. Abbreviations:* BMI = body mass index; zBMI = BMI z-scores; SE = standard error; lbCI = 95% confidence intervals lower bound; ubCI = 95% confidence intervals upper bound.

**eTable 10.** *The studies that examined intervention effects by socioeconomic status.*

| **Study** | **Measure of SES** | **Finding** |
| --- | --- | --- |
| *Alexandrou et al. (2023)* | Parental education | No evidence that the intervention effects were moderated by parental education. |
| *De Coen et al. (2012)* | Community SES level assessed by five indicators:  Number of births in underprivileged families  Proportion of pupils in primary school with a school delay  Rate of unemployment  Number of persons on welfare support  Number of underprivileged foreigners | Among children from low-SES communities, the intervention group showed a significant decrease in BMI Z-score compared to the low-SES control group; however, no significant effects were observed across the total sample. |
| *Enö Persson et al. (2018)* | Parental education | No evidence that parental education moderated the effect of the intervention. |
| *Iaia et al. (2017)* | Mother’s level of education | A significantly positive intervention effect was observed only among children whose mothers had a medium/high level of education compared to those with low education. |
| *Karssen et al. (2022)* | Parental education | The intervention appeared to be more effective for children of parents with a lower educational level. |

*Note. SES = Socioeconomic status.*

**eTable 11.** *The certainty of evidence scores based on the Grading of Recommendation, Assessment, Development, and Evaluation (GRADE) framework.*

| **Outcome** | **Description of effects** | **Total number of studies (study design)** | **Number of participants in included studies** | **Indirectness** | **Imprecision** | **Inconsistency across studies** | **Average RoB score** | **Other considerations** | **Certainty in evidence** |
| --- | --- | --- | --- | --- | --- | --- | --- | --- | --- |
| **zBMI** | 27 studies assessed BMI z-score, with 21 included in a meta-analysis (ES = 39), which showed a small but significant reduction in zBMI (SMD = -0.080; 95% CI: -0.133 to -0.028; n = 11,120) with moderate heterogeneity (I² = 46.78).  Six studies were excluded from the meta-analysis due to lack of a control group; their findings were mixed, with two showing significant effects and four showing no difference. | 27 (14 cluster RCT, 10 RCT, 3 experimental study) - Limited methodological concerns | 16,684 | Provide direct evidence to the clinical question at hand. | CI and precision estimates reported | Serious concerns | 4.5 - Fair | None | Moderate certainty ⊕ ⊕ ⊕ O |
| **Waist measures** | Eight studies assessed waist measures, of which seven studies assessed waist circumference and were included in a meta-analysis (ES = 11), showing a small but significant reduction (SMD = -0.186; 95% CI: -0.361 to -0.011; n = 3,906) with high heterogeneity (I² = 77.26).  One additional study measuring waist-hip ratio found no significant differences between groups. | 7 (4 cluster RCT, 3 RCT) - Very limited methodological concerns | 5,884 | Provide direct evidence to the clinical question at hand. | CI and precision estimates reported | No serious concerns | 4.8 - Fair | None | Moderate certainty ⊕ ⊕ ⊕ O |
| **BMI percentile** | 11 studies assessed BMI percentiles, with seven included in a meta-analysis (ES = 11), which found no significant intervention effect (SMD = 0.062; 95% CI: -0.040 to 0.163; n = 3,975) and high heterogeneity (I² = 57.56).  Four studies were excluded from the meta-analysis due to lack of a control group; one study aligned with the meta-analysis, while three reported significant reductions in BMI percentiles in the intervention group. | 11 (5 cluster RCT, 4 RCT, 2 experimental study) - Limited methodological concerns | 6,240 | Provide direct evidence to the clinical question at hand. | CI and precision estimates reported | Serious concern | 3.9 – Good | None | Moderate certainty ⊕ ⊕ ⊕ O |
| **Percentage body fat** | Three studies assessed percentage body fat and were included in a meta-analysis (ES = 4), which showed a modest but significant reduction in body fat percentage (SMD = -0.159; 95% CI -0.290, -0.028; n = 1,095) with low heterogeneity (I² < 0.001). | 3 (1 cluster RCT, 1 RCT, and 1 experimental) - Moderate methodological concerns | 1,525 | Provide direct evidence to the clinical question at hand. | CI and precision estimates reported | Serious concerns | 3.3 - Good | None | Low certainty  ⊕ O O O |
| **Fat-free mass index** | Three studies measuring fat-free mass index were included in a meta-analysis (ES = 3), which showed a small but significant positive effect (SMD = 0.170; 95% CI: 0.012 to 0.328; n = 919) with low heterogeneity (I² < 0.001). | 3 (3 RCT) - Very limited methodological concerns | 1,593 | Provide direct evidence to the clinical question at hand. | CI and precision estimates reported | No serious concern | 3.3 - Good | None | High certainty  ⊕ ⊕ ⊕ ⊕ |
| **Weight status category** | 14 studies assessed 19 weight status outcomes related to overweight and obesity prevalence. Among five studies on combined overweight/obesity prevalence, only one showed a significant reduction. Of six studies on obesity prevalence alone, two reported significant decreases. Nine studies on overweight prevalence found no significant changes. Overall, three studies showed reductions, while 17 reported no difference. | 14 (9 cluster RCT, 3 RCT, 2 experimental study) - Limited methodological concerns | 13,194 | Provide direct evidence to the clinical question at hand. | CI and precision estimates reported | Serious concerns | 4.6 - Fair | None | Low certainty  ⊕ O O O |
| **BMI** | 14 studies assessed intervention effect on BMI, of which 12 studies found no significant effect, while two reported significantly greater BMI reductions in the intervention group. | 13 (9 cluster RCT, and 5 RCT) - Very limited methodological concerns | 9,123 | Provide direct evidence to the clinical question at hand. | CI and precision estimates reported | No serious concern | 3.8 - Good | None | High certainty ⊕⊕⊕⊕ |
| **Skinfold measures** | Five studies reported six skinfold-related outcomes. Only one study found a significant intervention effect, and the remaining studies reported no significant differences. | 5 (3 RCT, and 2 cluster RCT) - Very limited methodological concerns | 4,296 | Provide direct evidence to the clinical question at hand. | CI and precision estimates reported | No serious concern | 4.2 - Fair | None | Moderate certainty ⊕ ⊕ O O |
| **Weight measures** | Eight studies reported body weight outcomes. Six studies measuring weight in kilograms found no significant post-intervention differences. Remaining two studies assessed weight z-scores, of which one study found no effect, while another reported significant reduction in weight z-scores in the group receiving both centre- and home-based interventions. | 8 (5 RCT and 3 cluster RCT) – Very limited methodological concerns | 2,948 | Provide direct evidence to the clinical question at hand. | CI and precision estimates reported | No serious concerns | 3.8 - Good | None | Moderate certainty ⊕ ⊕ ⊕ O |
| **Fat mass index** | Two studies measured fat mass index and reported no significant changes after intervention. | 2 (2 RCT) –  Very limited methodological concerns | 630 | Provide direct evidence to the clinical question at hand. | CI and precision estimates reported | No serious concerns | 3 - Good | None | Moderate certainty ⊕ ⊕ ⊕ O |
| Note. *BMI = Body mass index, ES = effect size; N = sample size; SMD = standardized mean difference; SE = standard error; lbCI =lower bound confidence interval; ubCI = upper bound confidence interval; I2 = heterogeneity* | | | | | | | | | |

**Figures**

**eFigure 1.** *Funnel plot of the effect sizes included in the meta-analysis of BMI z-scores.*


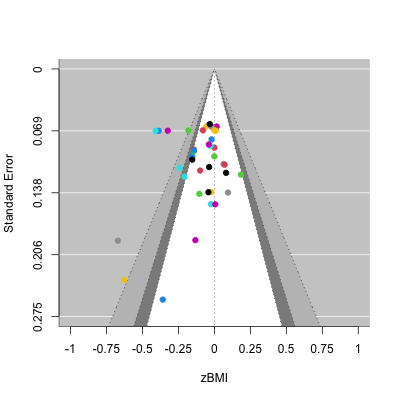


**eFigure 2.** *Funnel plot of the effect sizes included in the meta-analysis of waist circumference.*


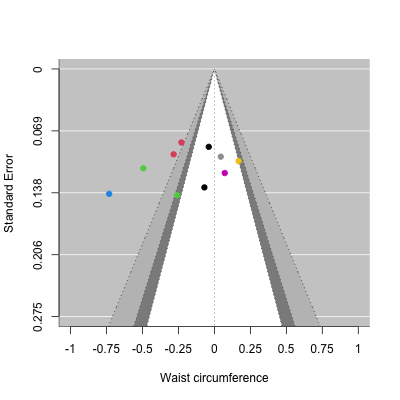


**eFigure 3.** *Funnel plot of the effect sizes included in the meta-analysis of BMI percentiles.*


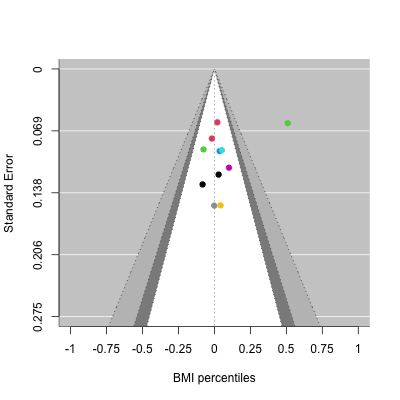


**eFigure 4.** *Funnel plot of the effect sizes included in the meta-analysis of percentage body fat.*


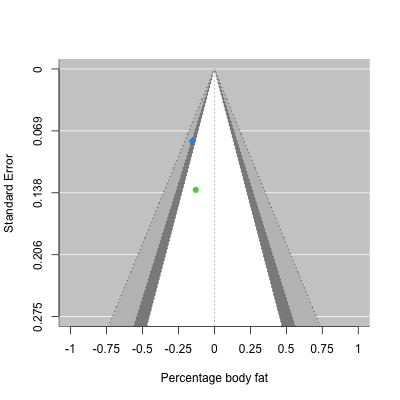


**eFigure 5.** *Funnel plot of the effect sizes included in the meta-analysis of fat-free mass index.*


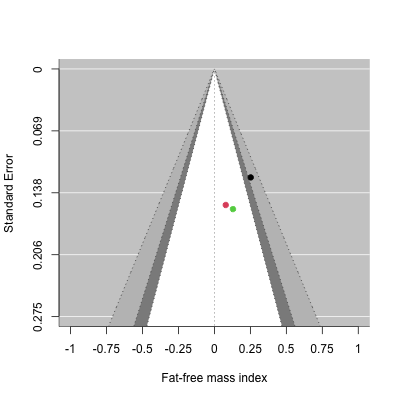


**eFigure 6.** *The scores on the National Institute of Health Quality Assessment Tool (Controlled Intervention Studies) for the studies included in the synthesis.*


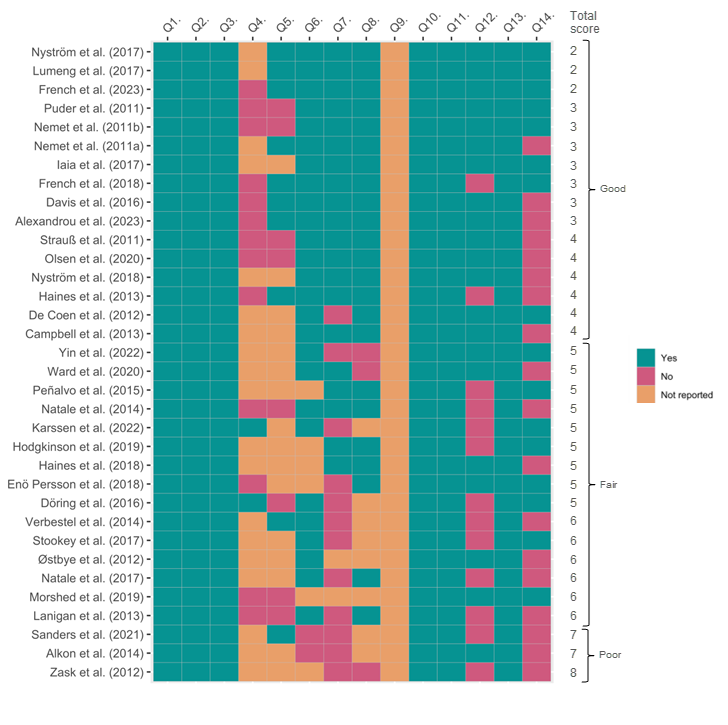
*Note.*

*Q1. Was the study described as randomized, a randomized trial, a randomized clinical trial, or an RCT?*

*Q2. Was the method of randomization adequate (i.e., use of randomly generated assignment)?*

*Q3. Was the treatment allocation concealed (so that assignments could not be predicted*

*Q4. Were study participants and providers blinded to treatment group assignment*

*Q5. Were the people assessing the outcomes blinded to the participants' group assignments*

*Q6. Were the groups similar at baseline on important characteristics that could affect outcomes (e.g., demographics, risk factors, co-morbid conditions)?*

*Q7. Was the overall drop-out rate from the study at endpoint 20% or lower of the number allocated to treatment?*

*Q8. Was the differential drop-out rate (between treatment groups) at endpoint 15 percentage points or lower?*

*Q9. Was there high adherence to the intervention protocols for each treatment group?*

*Q10. Were other interventions avoided or similar in the groups (e.g., similar background treatments)?*

*Q11. Were outcomes assessed using valid and reliable measures, implemented consistently across all study participants?*

*Q12. Did the authors report that the sample size was sufficiently large to be able to detect a difference in the main outcome between groups with at least 80% power?*

*Q13. Were outcomes reported or subgroups analyzed prespecified (i.e., identified before analyses were conducted)?*

*Q14. Were all randomized participants analyzed in the group to which they were originally assigned, i.e., did they use an intention-to-treat analysis?*

**eFigure 7.** *The scores on the National Institute of Health Quality Assessment Tool (Before-After [Pre-Post] Studies) for the studies included in the synthesis.*


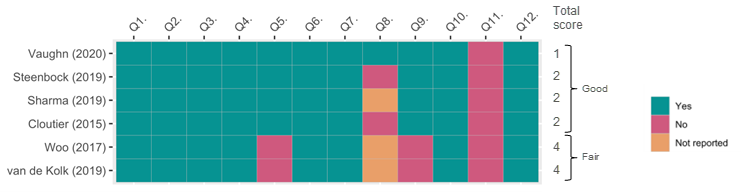
*Note.*

*Q1. Was the study question or objective clearly stated?*

*Q2. Were eligibility/selection criteria for the study population prespecified and clearly described?*

*Q3. Were the participants in the study representative of those who would be eligible for the test/service/intervention in the general or clinical population of interest?*

*Q4. Were all eligible participants that met the prespecified entry criteria enrolled?*

*Q5. Was the sample size sufficiently large to provide confidence in the findings?*

*Q6. Was the test/service/intervention clearly described and delivered consistently across the study population?*

*Q7. Were the outcome measures prespecified, clearly defined, valid, reliable, and assessed consistently across all study participants?*

*Q8. Were the people assessing the outcomes blinded to the participants' exposures/interventions?*

*Q9. Was the loss to follow-up after baseline 20% or less? Were those lost to follow-up accounted for in the analysis?*

*Q10. Did the statistical methods examine changes in outcome measures from before to after the intervention? Were statistical tests done that provided p values for the pre-to-post changes?*

*Q11. Were outcome measures of interest taken multiple times before the intervention and multiple times after the intervention (i.e., did they use an interrupted time-series design)?*

*Q12. If the intervention was conducted at a group level (e.g., a whole hospital, a community, etc.) did the statistical analysis take into account the use of individual-level data to determine effects at the group level?*
